# Common mode voltage of three phase split source inverters for solar pumping applications

**DOI:** 10.1038/s41598-026-59032-6

**Published:** 2026-08-26

**Authors:** Mostafa Wageh Lotfy, Safaa Ibrahim, Arafa S. Mansour, Mohamed. A. Ghalib, Atef M. Mansour, Abdel-Gawad A. Abdel-Samei

**Affiliations:** 1 Dept. of Process Control Technology, Faculty of Technology and Education, Beni-Suef University, Beni-Suef, Egypt; 2 Department of Electronics Technology, Faculty of Technology and Education, Beni-Suef University, Beni-Suef, Egypt; 3 Electrical Engineering Department, Faculty of Engineering, Beni-Suef University, Beni-Suef, 62511 Egypt; 4 Power Electronics and Energy Conversion Department, Electronics Research Institute, Cairo, 12622 Egypt

**Keywords:** Three-phase split-source inverter (SSI), Solar pumping system, Space vector PWM, Leakage current, Common-mode voltage, Energy science and technology, Engineering

## Abstract

Low-voltage photovoltaic (PV) systems often employ a cascaded power conversion structure comprising a separate boost converter and inverter, leading to increased complexity and reduced overall efficiency. The split-source inverter (SSI) topology addresses these limitations by combining voltage boosting and inversion functionalities within a single-stage architecture, offering improved integration and reduced component count. This paper proposes a space vector modulation (SVM) strategy for SSIs and compares it against a modified SVM (MSVPWM) approach designed to mitigate high-frequency common-mode voltage (CMV). A detailed analysis is carried out focusing on CMV characteristics, leakage current behavior, and total harmonic distortion (THD) in line-to-line voltages. Experimental and simulation-based comparisons with conventional modulation methods are provided to validate the effectiveness of the MSVPWM approach, demonstrating notable improvements in electromagnetic performance and output waveform quality. In particular, the MSVPWM technique outperforms the conventional SVPWM method, achieving a 7.6% reduction in voltage THD and a 66.7% reduction in CMV. These improvements confirm the suitability of the MSVPWM method for PV powered pumping systems and other applications that demand high power quality.

## Introduction

With the growing demand for sustainable energy, solar-powered water pumping systems have emerged as a viable and environmentally friendly alternative to conventional irrigation methods, especially in remote and off-grid areas. Among the key components of these systems is the power inverter, which plays a crucial role in converting the variable DC output from solar panels into a stable AC supply suitable for driving three-phase motors commonly used in water pumps^1^.

Split-source inverters (SSIs), a subset of impedance-source inverters, have gained significant attention in recent years due to their single-stage power conversion, voltage boost capability, and enhanced reliability. Their ability to handle a wide input voltage range makes them particularly well-suited for photovoltaic (PV) applications, where input conditions can vary due to changes in sunlight and temperature^2^. However, one of the critical challenges in the operation of three-phase SSIs is the generation of common-mode voltage (CMV), which can adversely affect the performance and longevity of the motor and other connected equipment. High CMV levels can lead to increased electromagnetic interference (EMI), bearing currents, and insulation stress in motor windings, potentially reducing system efficiency and reliability. SSIs introduced as a variation of impedance-source inverters, provide voltage boosting and inversion in a single-stage topology. This is particularly advantageous for PV applications where a wide voltage variation exists due to environmental conditions. Studies such as^3–6^ have highlighted the benefits of SSIs in improving power conversion efficiency and reducing component stress. However, limited attention has been given to their CMV characteristics in three-phase configurations.

Various PWM techniques have been proposed to enhance the performance of quasi-Z-source inverters (qZSIs)^7–15^. These methods have been classified, reviewed, and compared in the existing literature based on factors such as modulation range, boosting capability, voltage gain, capacitor voltage stress, inductor current ripple, voltage/current stress on power devices, inverter efficiency, and ease of implementation^16,17^. However, due to the inherent nature of PWM algorithms, all of these techniques produce undesirable CMV. In PV systems, where there is significant parasitic capacitance between the PV array and the grounded frame, the magnitude of CMV becomes a critical factor in assessing leakage current^18^. According to safety standards such as IEC 62,109–2, this leakage current must be minimized. To date, the CMV generated by both conventional and advanced PWM strategies for qZSIs has not been thoroughly explored or discussed in the literature. Only the study in^19^ provides a comparison of several conventional PWM schemes for qZSIs with respect to CMV magnitude. However, this comparison focuses on a limited CMV bandwidth rather than evaluating its root mean square (RMS) value.

One limitation of the aforementioned topologies and modulation strategies is their reliance on a high input voltage to meet grid-voltage standards. Conventional solutions typically use a two-stage architecture, consisting of a boost converter followed by an inverter, to raise the DC-link voltage^20^. Alternatively, several topologies have been developed to eliminate the need for a separate boost stage^21^. The impedance-source inverter (ZSI), introduced in^22^, incorporates the boosting function directly into the inverter stage, reducing the number of switching components. For transformerless PV applications^23^, proposed a modified ZSI topology coupled with a new modulation strategy. While this approach effectively mitigates leakage current, it is constrained by a limited modulation index and a high boost ratio. In contrast^24^, introduces a carrier-based modulation scheme for the ZSI that maintains a constant common-mode voltage (CMV). However, this solution requires a four-leg inverter, which increases the number of active components in the power conversion system.

Recently, the split-source inverter (SSI) introduced in^25^ has emerged as a promising topology that combines the boost and inverter stages into a single DC-AC power stage. The SSI utilizes a conventional inverter bridge along with three additional diodes. Unlike the Z-source inverter (ZSI), the SSI does not require shoot-through states and uses fewer passive components. Several topological modifications of the SSI have been proposed in^26–28^. In^29,30^, the authors introduce a discontinuous PWM (DPWM) technique aimed at reducing CMV in SSIs. However, this method does not achieve substantial CMV reduction. Additionally, it introduces low-frequency ripple in the input current and DC-link voltage. Therefore, the development and analysis of effective modulation strategies for CMV reduction in split-source inverters remain open research areas requiring further investigation.

This study focuses on the analysis and mitigation of CMV in three-phase SSIs tailored for solar water pumping applications. By investigating the root causes of CMV generation and evaluating various modulation techniques and design approaches, the research seeks to enhance the electromagnetic compatibility and overall efficiency of SSI-based solar pumping systems. A comprehensive evaluation is carried out, focusing on leakage current, total harmonic distortion (THD) of the output voltage, and the maximum achievable modulation index. Additionally, this work expands upon the preliminary findings presented in the conference paper^31^. The paper is organized as follows: Section “Analysis of common-mode voltage in split-source inverter topologies” reviews the operating principles of the SSI and discusses the concepts of CMV and leakage current. Section “Space Vector PWM Techniques” presents a comparative analysis supported by experimental validation of the theoretical results. Finally, Section “Results and analysis” concludes the paper by summarizing the main contributions and advantages of the proposed strategies.

## Analysis of common-mode voltage in split-source inverter topologies

**Fig. 1:**
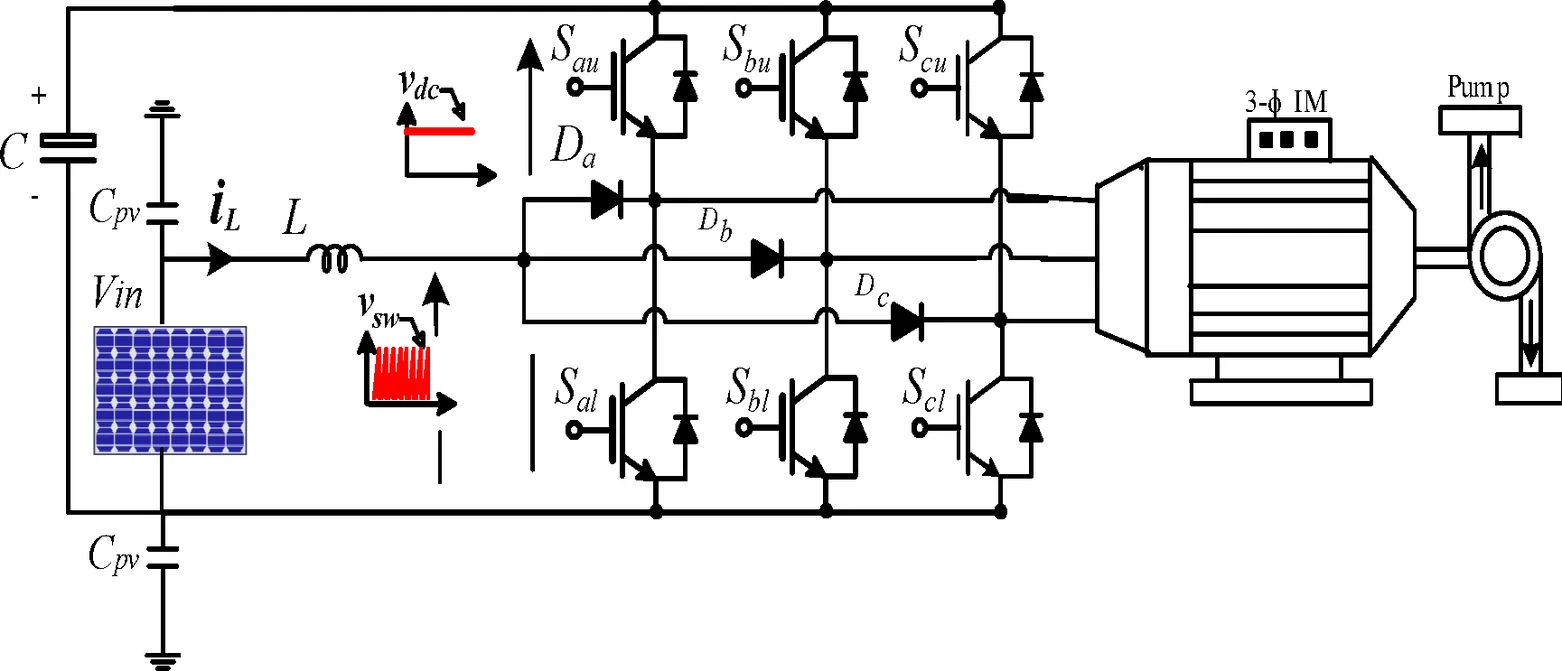
3-Φ spilt-source inverter (SSI).

Three-phase SSIs have become increasingly popular for efficient DC-AC conversion, particularly in renewable energy applications. This topology enables voltage boosting from DC sources while enhancing output quality, as illustrated in Fig. 1.

The split-source inverter topology introduced in^25^ is illustrated in Fig. 1. Similar to a conventional full-bridge inverter, the SSI operates in eight distinct switching states. Table 1 outlines these inverter states along with their corresponding voltage vectors in the $\alpha \beta$-coordinate frame. When at least one of the lower switches is turned ON corresponding to states ${V}_{0}$ to ${V}_{6}$ the inductor *L* stores energy from the input source. Conversely, when all the upper switches are turned *ON*, representing state ${V}_{7}$, the inductor *L* transfers its stored energy to the capacitor *C*. The duty cycle *D* is defined as $D-1-\frac{{t}_{7}}{{T}_{x}}$, where ${t}_{7}$ is the dwell time of state ${V}_{7}$ and ${T}_{s}$ is the total switching period. The voltage gain of the inverter can thus be expressed as:

**Table 1:** Inverter switching states, and common-mode voltage levels.

Stat	A	B	C	${V}_{\alpha }$	${V}_{\beta }$	*V*_cm_
[000]	0	0	0	0	0	0
[001]	0	0	1	$\frac{2}{3}$	0	$\frac{{V}_{C}}{3}$
[010]	0	1	0	$\frac{1}{3}$	$\frac{\sqrt{3}}{3}$	$\frac{{2V}_{C}}{3}$
[011]	0	1	1	$\frac{-1}{3}$	$\frac{\sqrt{3}}{3}$	$\frac{{V}_{C}}{3}$
[100]	1	0	0	$\frac{-2}{3}$	0	$\frac{{2V}_{C}}{3}$
[101]	1	0	1	$\frac{-1}{3}$	$\frac{-\sqrt{3}}{3}$	$\frac{{V}_{C}}{3}$
[110]	1	1	0	$\frac{1}{3}$	$\frac{-\sqrt{3}}{3}$	$\frac{{2V}_{C}}{3}$
[111]	1	1	1	0	0	${V}_{C}$

1 $$\frac{{V}_{C}}{{V}_{\mathrm{i}\mathrm{n}}}=\frac{1}{1-D}$$

Here, $\frac{{t}_{7}}{{T}_{2}}$ represents the normalized dwell time of vector ${V}_{7}$, denoted as ${d}_{7}$.

Furthermore, the CMV produced by the inverter is a key factor in exciting leakage currents. Therefore, accurately quantifying the CMV generated by the inverter is crucial, as described in this section. Referring to the circuit shown in Fig. 1, the voltage between the negative input terminal *N* and the grounded neutral point n can be derived using Kirchhoff’s voltage law as:

2 $${v}_{Nn}={v}_{xN}+{v}_{xn}=0$$

where *x=a,b,c*. By summing all instances of Eq. (2), the voltage becomes:

3 $${v}_{Nn}=\frac{1}{3}\sum_{x=a}^{c} \left({v}_{xN}+{v}_{xn}\right)$$

Similarly, the voltage between the positive input terminal *P* and the grounded neutral *n* can be expressed as:

4 $${v}_{Pn}=\frac{1}{3}\sum_{x=a}^{c} \left({v}_{PN}-{v}_{PN}+{v}_{xP}+{v}_{xn}\right)$$

In a balanced three-wire system, the sum of the inverter line-to-neutral voltages is zero:

5 $${v}_{an}+{v}_{bn}+{v}_{cn}=0$$

Moreover, the voltage ${v}_{x}P$ can be expressed in terms of the negative terminal voltage as:

6 $${v}_{xP}={v}_{xN}+{v}_{PN}$$

Substituting equations (5) and (6) into (2) and (3), the common-mode voltage can be derived as:

7 $${V}_{cm}=\frac{{v}_{Nn}+{v}_{Pn}}{2}=\frac{{v}_{aN}+{v}_{bN}+{v}_{cN}}{3}+\frac{{v}_{PN}}{2}$$

Assuming the input voltage remains constant, the CMV can be shifted by $\frac{{V}_{t}}{2}$. Consequently, the common-mode voltage generated by the inverter is defined in Eq. (8), where ${S}_{1},{S}_{2}$, and ${S}_{3}$ denote the switching states of the inverter:

8 $${V}_{cm}=\frac{{V}_{C}\left({S}_{1}+{S}_{2}+{S}_{3}\right)}{3}$$

**Fig. 2:**
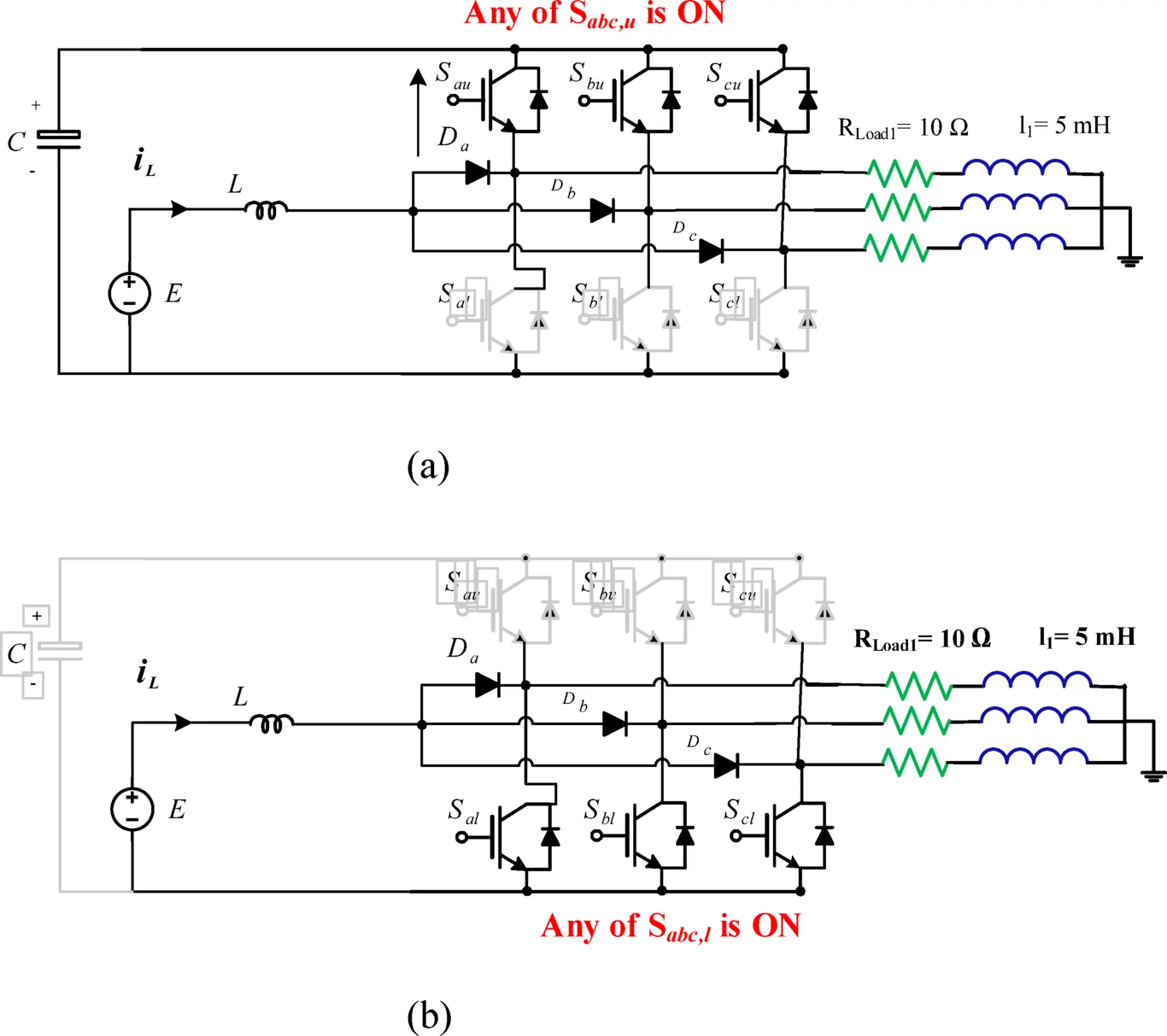
The potential states of the three-phase SSI while charging L and discharging L.

As illustrated in Fig. 2, inductor *L* begins charging whenever at least one of the three reference signals (${v}_{abc}^{*}$) is lower than the carrier signal. This behavior can be better understood by introducing a virtual envelope defined by the minimum value of the reference signals, min(${v}_{abc}^{*}$), Accordingly, the SSI operates as follows: when min(${v}_{abc}^{*}$), is below the carrier signal, *L* charges (Fig. 2a), whereas when min(${v}_{abc}^{*}$), rises above the carrier signal, *L* discharges and the dc-link capacitor *C* charges (Fig. 2b).

The dynamic behavior of the input inductor current and the DC-link capacitor voltage can be described by the following relations^5^:

9 $$\frac{{di}_{L}}{dt}={V}_{DC}$$

10 $$C\frac{{d}_{Vc}}{dt}={i}_{C}$$

where *C* is the capacitance of the *DC, L* is the inductance of the input inductor.

Three-phase SSIs offer high efficiency and the ability to provide higher output voltages, making them ideal for renewable energy applications. They reduce component count, lower costs, and improve reliability. However, they face challenges such as complex control requirements, voltage stress on switches, and potential additional filtering needs. These inverters are widely used in photovoltaic systems, electric vehicle charging, uninterruptible power supplies, and variable speed drives^29,30^.

The discharging *L* and charging *C* can be expressed as follows:

11 $$C\frac{{d}_{Vc}}{dt}={i}_{C}-{i}_{L}$$

12 $$L\frac{{di}_{L}}{dt}={V}_{DC}-{V}_{C}$$

## Space vector PWM techniques

For each of the eight switching states of the common VSI, the space-vector $\overrightarrow{V}$ may be created by:

13 $$\overrightarrow{V}=2/3( {V}_{an}+{V}_{bn}{e}^{j2\pi /3}+{V}_{cn}{e}^{j4\pi /3})$$

In three-phase voltage source inverters (VSIs) with space vector modulation (SVM), eight voltage vectors are available, six active and two zero vectors. Each vector corresponds to a specific instantaneous CMV, providing insight into inverter behavior^25^. In conventional SVPWM, the desired reference vector (${\overrightarrow{V}}^{*}={\mathrm{Me}}^{\mathrm{j}\upvartheta }$) is synthesized by combining adjacent active vectors (e.g., $\overrightarrow{{V}_{1}}$ and $\overrightarrow {{V_{2} }}$ when ${\overrightarrow{V}}^{*}$ lies in sector A1) along with zero vectors.

This process relies on the principle of volt-second balancing, where the average inverter voltage over a switching period ${T}_{S}$ equals the reference vector. By accurately calculating the duty cycles of each vector, the inverter achieves an output voltage that closely follows the reference, minimizing distortion and switching losses^5^.

Through precise timing control, SVPWM delivers high-quality, sinusoidal output, making it suitable for renewable energy systems, electric vehicles, and industrial drives. The method not only improves efficiency but also enhances system stability and reliability.

14 $${{T}_{s}\overrightarrow{V}}^{*}={T}_{1}\overrightarrow{{V}_{1}}+{T}_{2}\overrightarrow{{V}_{2}}+{T}_{z}\overrightarrow{{V}_{z}}$$

where ${T}_{1},{T}_{2},{T}_{z}$ are the active period’s application time and zero vectors ${V}_{1},{V}_{2},{V}_{z}$ respectively, which are calculated by:

15 $$\begin{gathered} T_{1} = T_{s} M\sin (\pi /3 - \vartheta ), \hfill \\ T_{2} = T_{s} M\sin (\vartheta ), \hfill \\ T_{z} = T_{s} - T_{1} - T_{2} \hfill \\ \end{gathered}$$

${T}_{z}$ is distributed equally between the zero vectors ${V}_{0}$ and ${V}_{7}$ with ${T}_{0}$ and ${T}_{7}$ in the traditional SVPWM. Therefore,

16 $${T}_{0}={T}_{7}=0.5{T}_{z}=(1-M\mathrm{sin} (\pi /3-\vartheta ))$$

For the full linear region in the range of $0\le M\le 1$, Eq. (16) is accurate.

### Modified SVPWM (MSVPWM) Technique for SSI

**Fig. 3:**
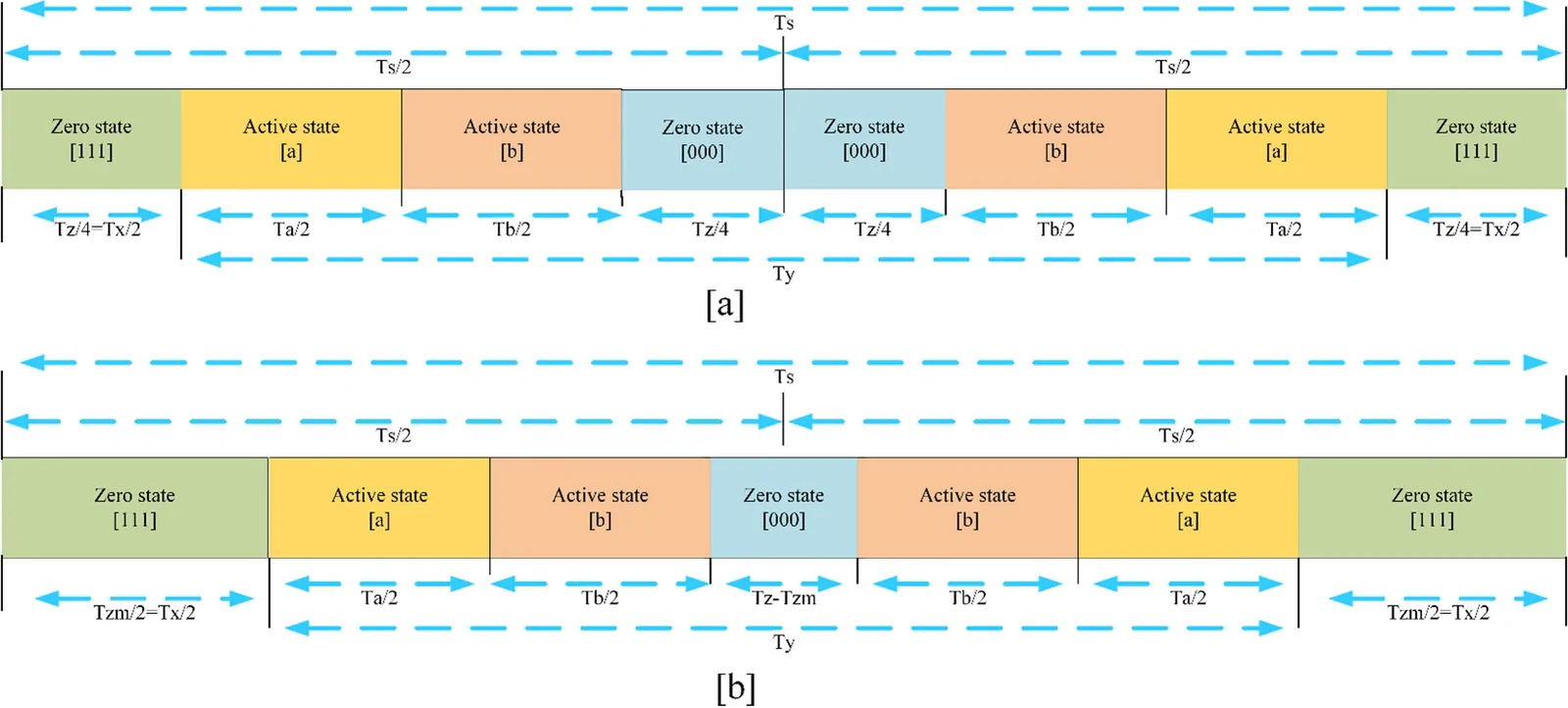
Switching patterns within a sector: (**a**) conventional SVPWM scheme and (**b**) modified MSVPWM scheme, where *T*_x_ denotes the inductor discharging time per switching cycle and *T*_y_​ represents the inductor charging time per switching cycle.

The presence of low-frequency components in the inductor current and inverter voltage under MSVPWM can be effectively mitigated by fixing the duty cycle *D*. This adjustment is achieved by revisiting the switching pattern of the conventional SVPWM scheme, as illustrated in Fig. 3a, where ${T}_{s}$ is the sampling period, ${T}_{a}$ and ${T}_{b}$ represent the durations of the active states, and ${T}_{z}$ corresponds to the total zero-state duration^13^. The key idea lies in redistributing the zero-state time without altering the active-state durations.

To achieve this, the inductor discharging time ${T}_{x}$ is set to the minimum zero-state duration, ${T}_{zm}$^26^, expressed as:

17 $${T}_{x}={T}_{zm}={T}_{s}\{1-2M\mathrm{sin} (\pi /6)\}$$

Within each sector, shown in Fig. 3b, the remaining zero-state duration is allocated to the complementary zero state. By enforcing ${T}_{x}={T}_{zm,}$ the required biasing is automatically established, as demonstrated in the simulation in Fig. 19. The modified reference signals ensure that the lower virtual envelope remains constant, thereby fixing the duty cycle *D* at *M*. Consequently, the normalized inverter voltage and the output fundamental peak phase voltage are obtained as^30^:

18 $$\begin{array}{c}\frac{{V}_{inv}}{{V}_{dc}}=\frac{1}{1-M}\\ \frac{{V}_{\phi }}{{V}_{dc}}=\frac{1}{\sqrt{3}(1-M)}\end{array}$$

Compared with conventional continuous modulation techniques, the MSVPWM strategy offers significant advantages. It requires only three reference signals and minimizes the number of commutations. Moreover, the effective switching frequency of the upper switches ( ${S}_{au},{S}_{bu},{S}_{cu}$ ) becomes two-thirds of the carrier frequency, while for the lower switches ( ${S}_{al},{S}_{bl},{S}_{cl}$ ) it equals the carrier frequency. As a result, the total number of switching transitions is substantially reduced relative to traditional modulation strategies, leading to improved efficiency and lower switching losses.

## Results and analysis

To conduct an in-depth analysis of the proposed inverter topology, the MATLAB-SIMULINK® environment was employed, using the system parameters specified in Table 2 to ensure a thorough and accurate evaluation. The simulated configurations of three-phase inverter topologies were specifically designed to deliver power to a three-phase inductive load, modeled as a three-phase induction motor, which closely reflects practical industrial applications. The entire system was energized by a low-voltage DC power source operating at 90 V.this voltage level was chosen to emulate the output of a PV-panel under realistic operating conditions, thereby assessing the inverter compatibility with renewable energy systems. To achieve accurate switching control and optimal modulation performance, a leading-edge sawtooth carrier waveform with a high frequency of 15 kHz was utilized. This approach ensured precise pulse-width modulation (PWM), minimizing harmonic distortion and enhancing the inverter’s dynamic response.

**Table 2:** Elements used to simulate the analyzed topology.

Element	Split-source inverter (SSI)
*M*	0.**7**
${F}_{I}$	15,000 Hz
${F}_{O}$	50 Hz
Capacitances and Inductance	${C}_{1},{C}_{2}=470\mu$ F, ${L}_{1}=3$ mH
dc voltage	90 V

For the SSI configuration, the DC-link circuit was carefully designed to include a 3 mH inductor and a 470 µF capacitor. This selection was made to balance transient response and steady-state stability, thereby enhancing energy conversion efficiency and maintaining a stable output under dynamic load variations.

The simulation results presented in Figs. 4, 5, 6, 7, 8, 9, 10, 11, 12, 13, 14, 15, 16, 17, 18, 19, 20, 21, 22 and 23 offer a detailed analysis of the operational performance of the SSI under SVPWM and MSVPWM strategies. These waveforms collectively highlight the dynamic behavior, energy conversion efficiency, and stability of the inverter when supplying power to a three-phase inductive load. Figures 4 and 5 present the simulated phase voltage waveforms obtained using SVPWM and MSVPWM strategies. demonstrating the inverter ability to produce balanced and sinusoidal output voltages in accordance with the applied switching strategy.

**Fig. 4:**
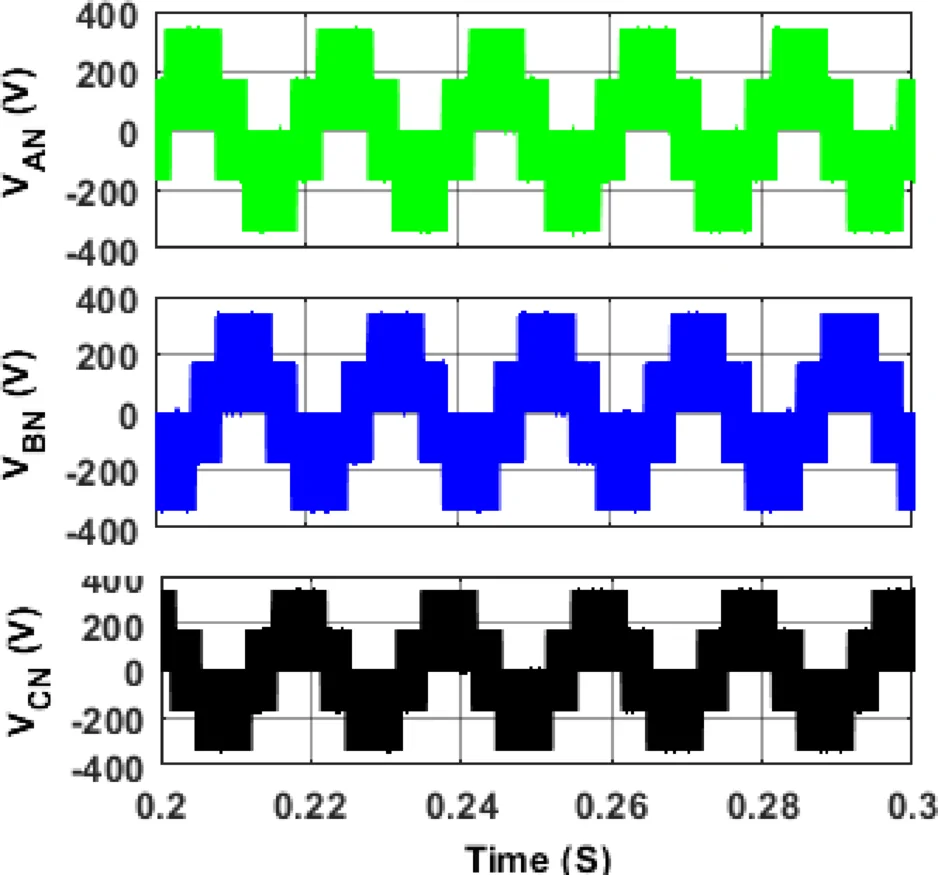
Output phase voltage for SVPWM.

**Fig. 5:**
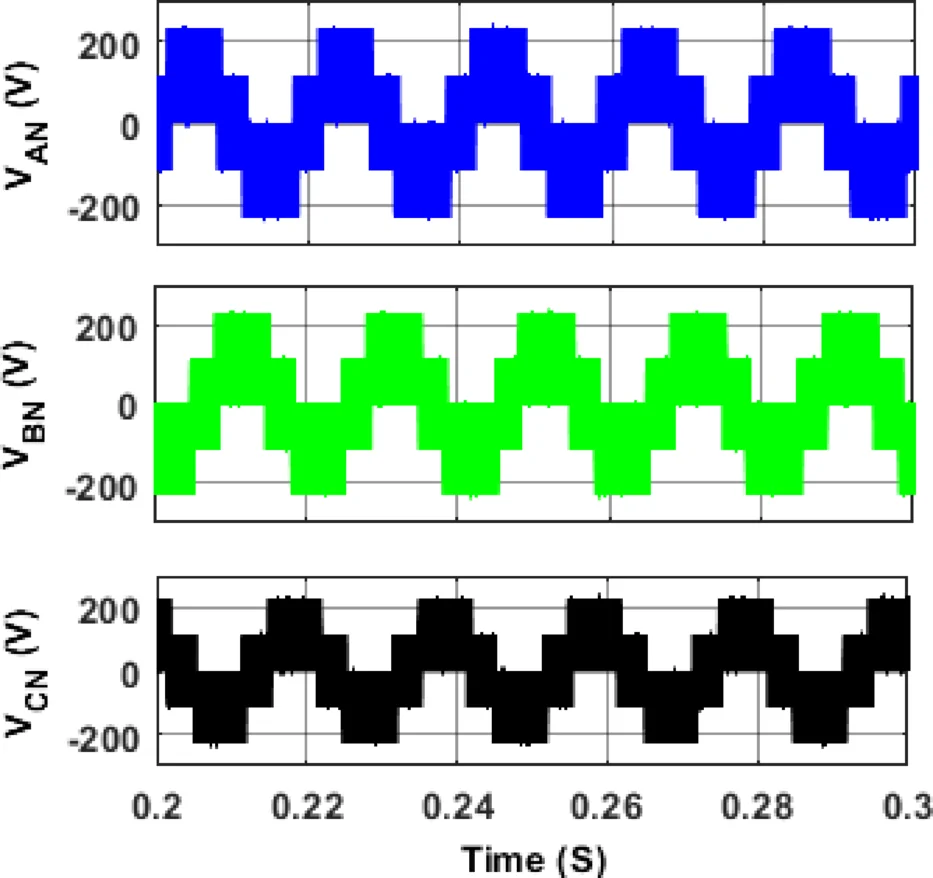
Output phase voltage for MSVPWM.

**Fig. 6:**
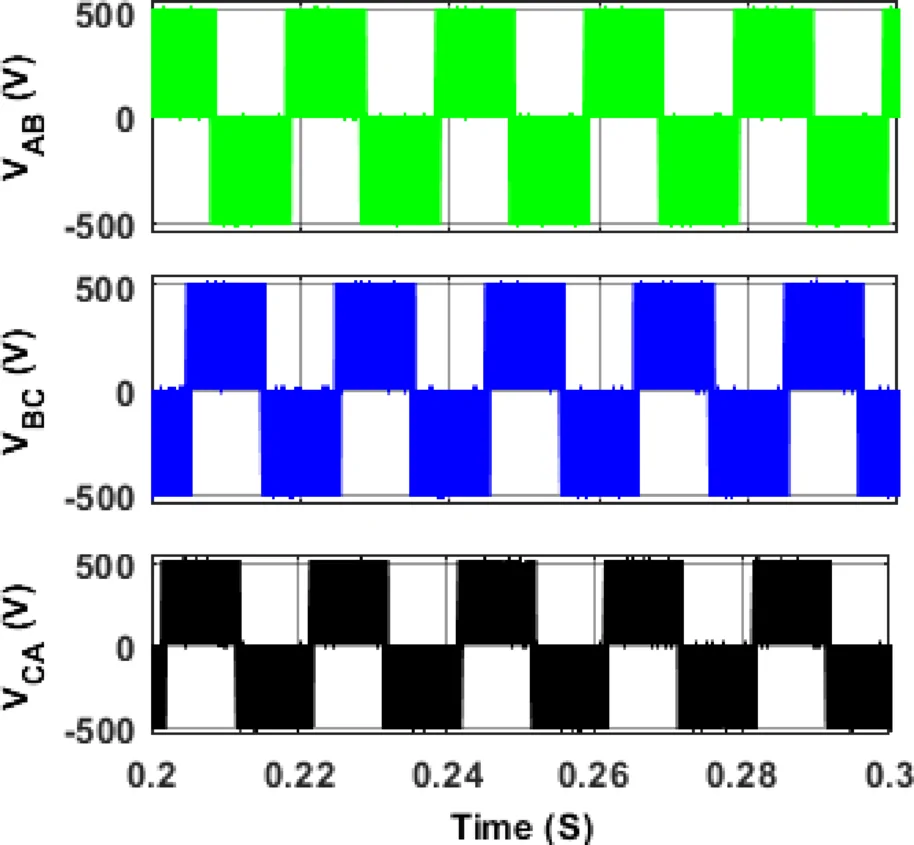
Output Line-Line voltage for SVPWM.

**Fig. 7:**
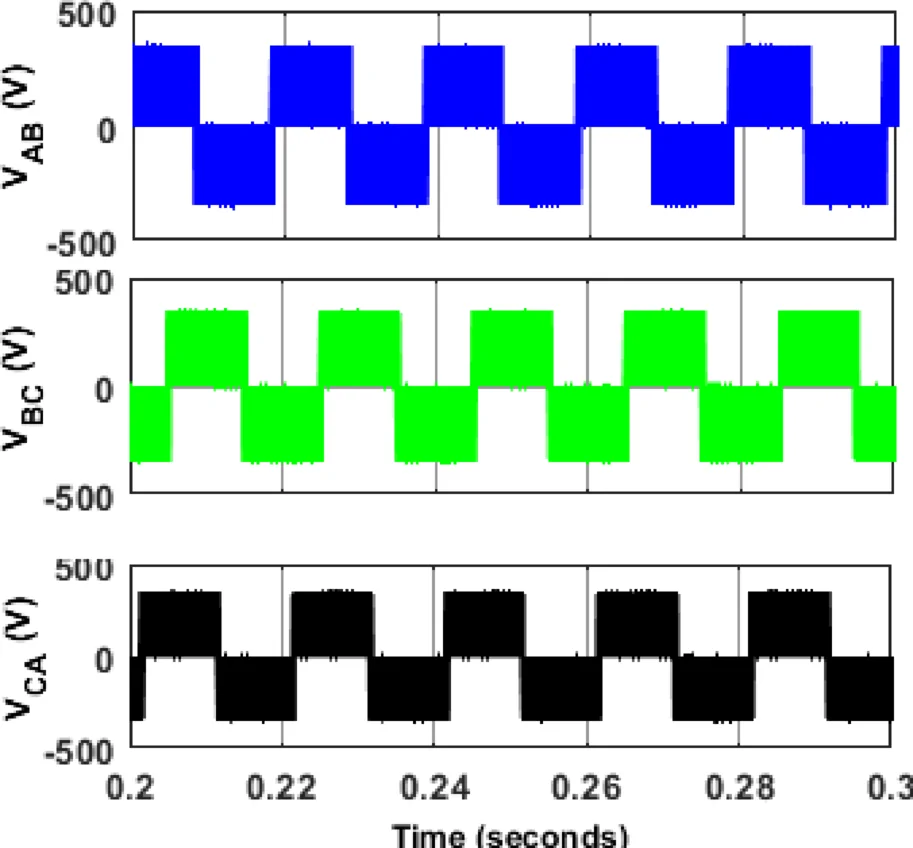
Output Line-Line voltage for MSVPWM.

**Fig. 8:**
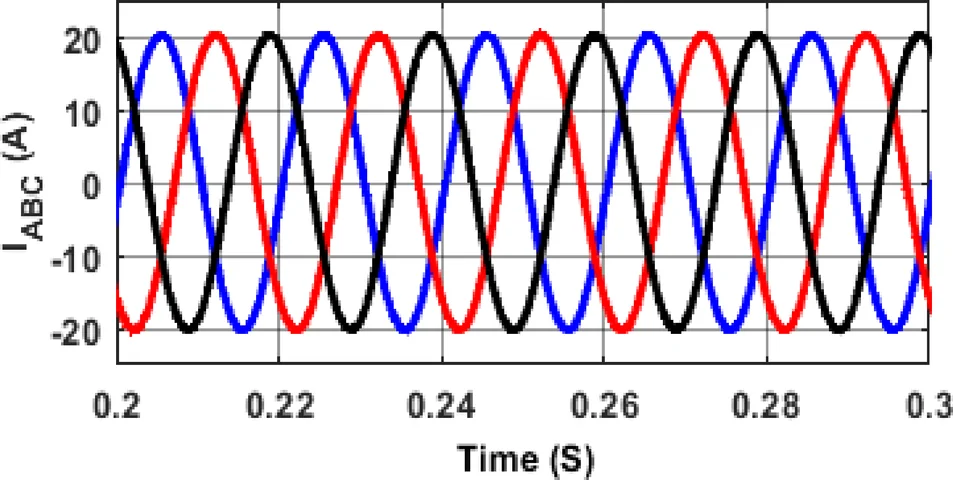
Three phase output current for SVPWM.

**Fig. 9:**
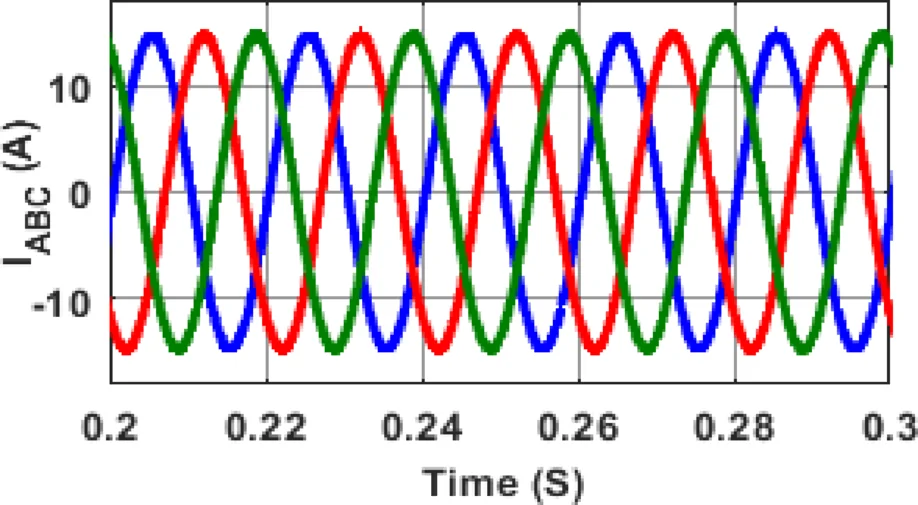
Three phase output current for MSVPWM.

**Fig. 10:**
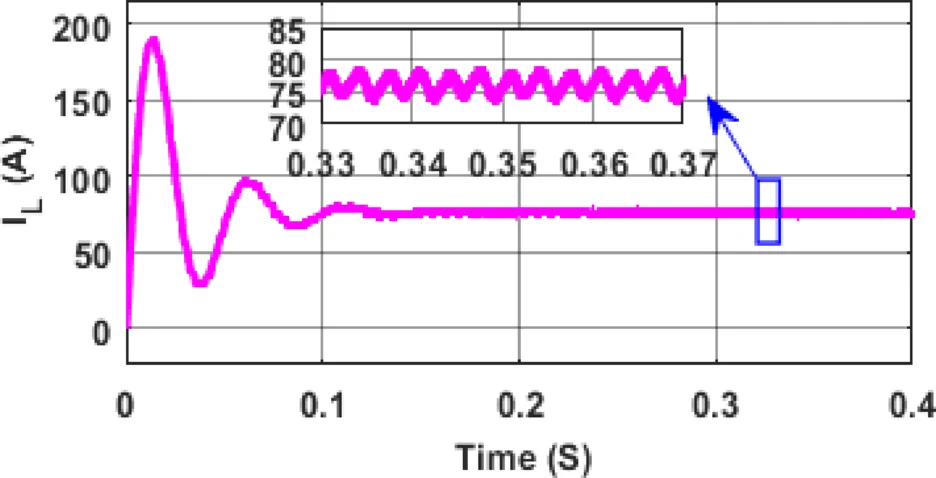
Inductor current waveform of the SSI under SVPWM.

**Fig. 11:**
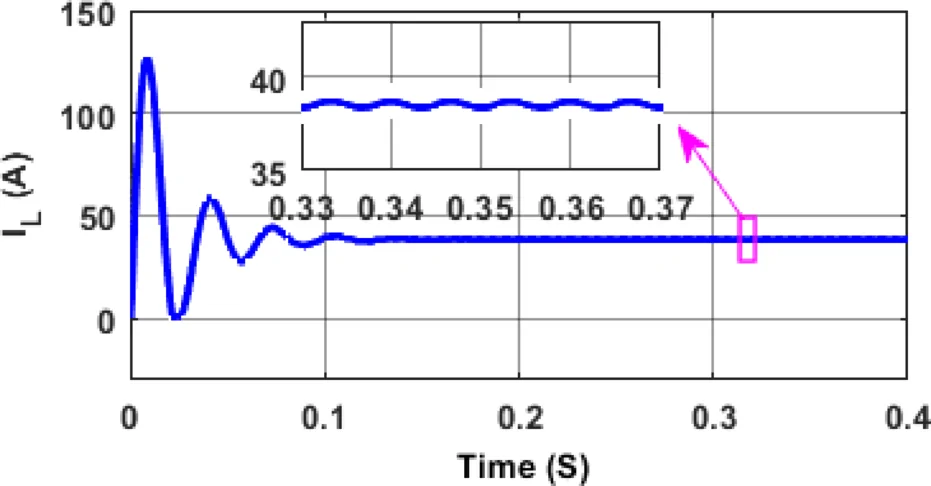
Inductor current waveform of the SSI under MSVPWM.

**Fig. 12:**
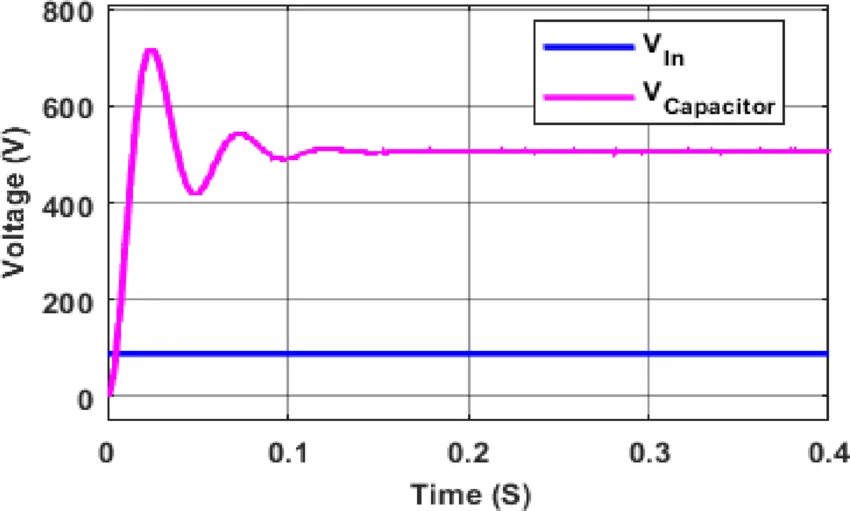
Capacitor voltage waveform of the SSI under SVPWM.

**Fig. 13:**
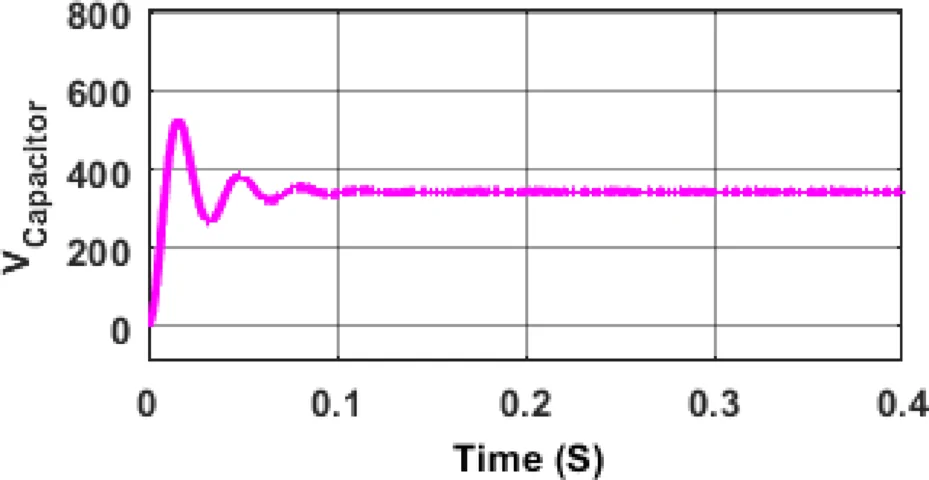
Capacitor voltage waveform of the SSI under MSVPWM.

Figures 6 and 7 illustrate the corresponding line voltage waveforms obtained using SVPWM and MSVPWM strategies, showcasing the voltage difference between inverter output terminals and confirming the consistency of the three-phase voltage system. Figures 8 and 9 display the output line current waveform, reflecting the current response of the system under-load and indicating the inverter capability to maintain continuous and stable current delivery. Figures 10 and 11 show the inductor current waveform, providing insights into the energy storage and transfer characteristics of the SSI boost network, which is essential for maintaining DC-link voltage stability. Figures 12 and 13 present the capacitor voltage waveform for the SVPWM and MSVPWM methods, capturing the voltage dynamics across the SSI’s capacitive components and highlighting their role in filtering and voltage regulation.

**Fig. 14:**
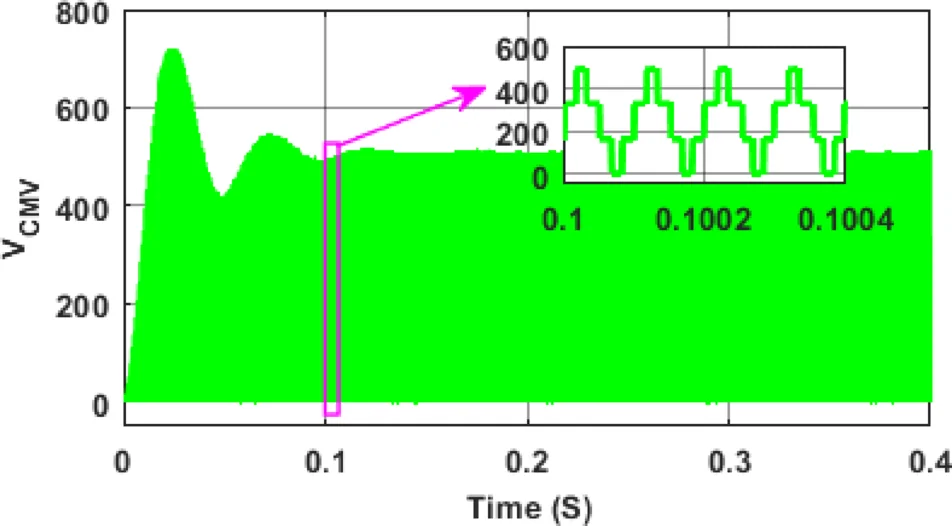
CMV waveform of the SSI under SVPWM.

**Fig. 15:**
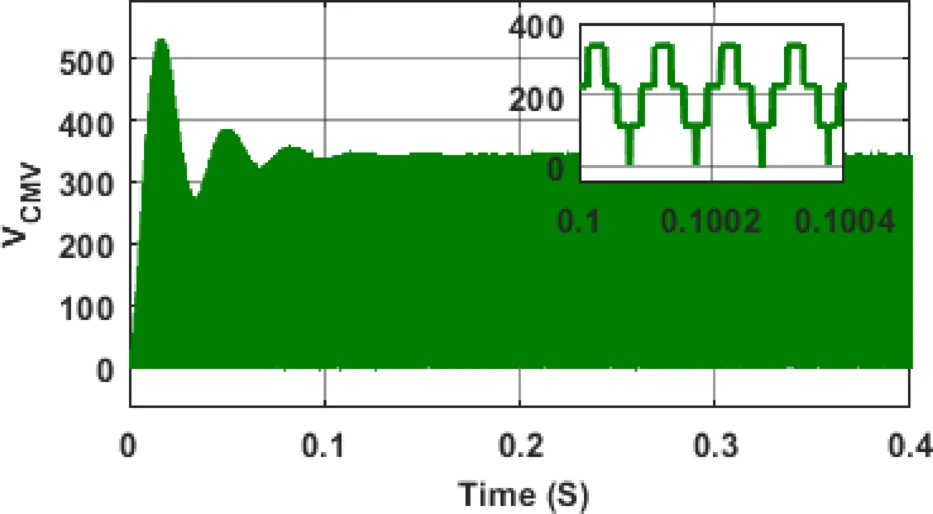
CMV waveform of the SSI under MSVPWM.

Together, these simulation waveforms validate the design robustness and operational reliability of the SSI topology. The consistent and smooth profiles of the voltages and currents confirm that the inverter is well-suited for applications in renewable energy systems, such as PV integration, and in electric motor drives, where precise power conversion and stable operation are critical, Figs. 14 and 15 present the corresponding simulation results, highlighting the system behavior under SVPWM and MSVPWM control strategies. As shown in Fig. 15, the CMV is reduced compared to the SVPWM method.

**Fig. 16:**
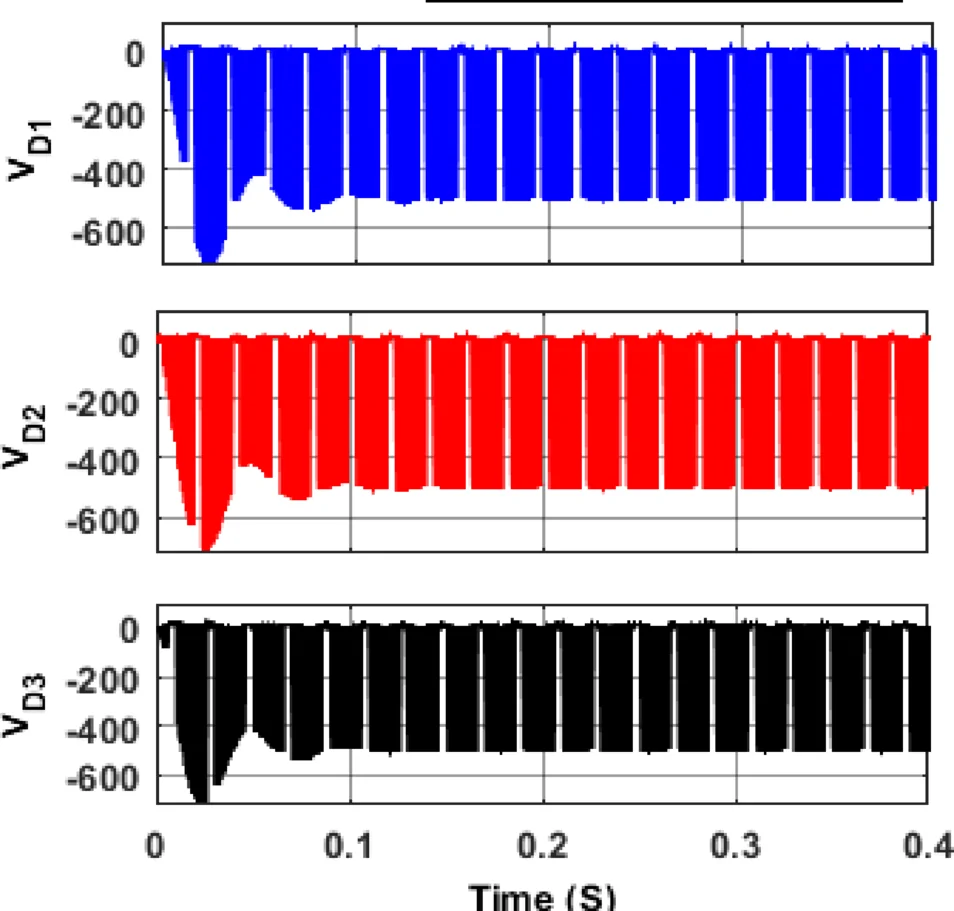
Diode voltage waveform of the SSI under SVPWM.

**Fig. 17:**
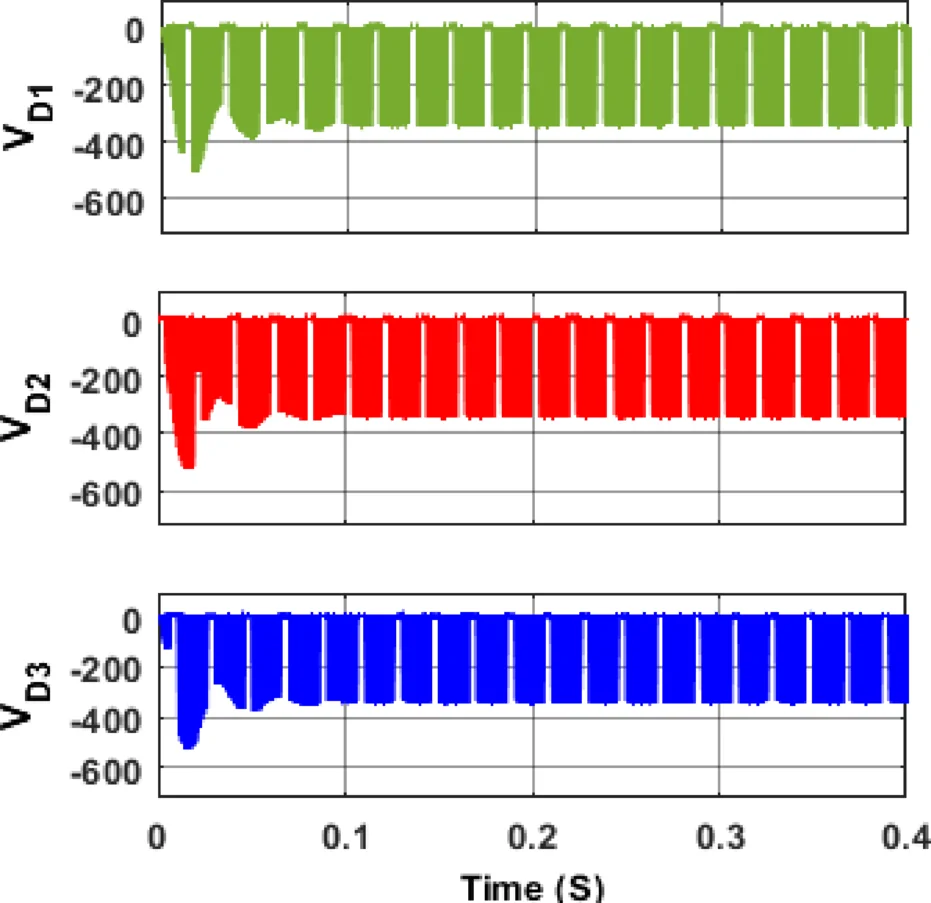
Diode voltage waveform of the SSI under MSVPWM.

**Fig. 18:**
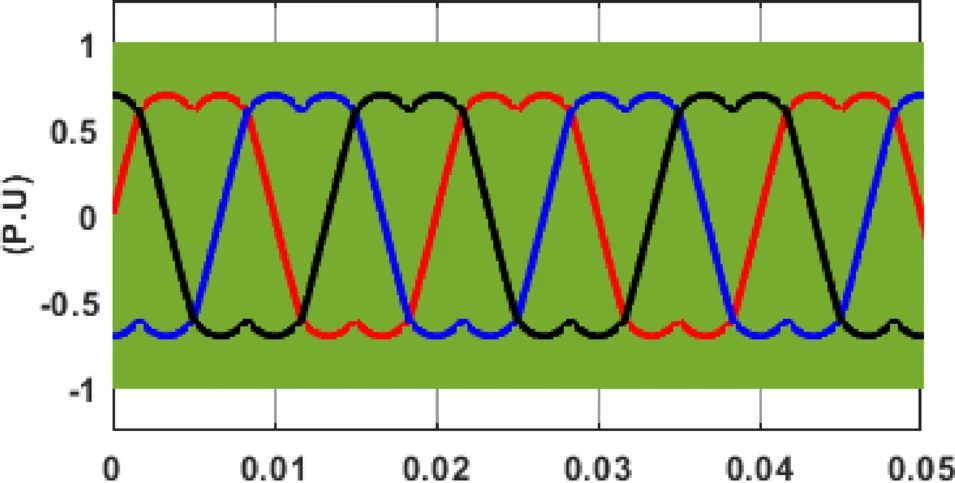
Three-phase modulating signal waveforms for the SVPWM schemes.

**Fig. 19:**
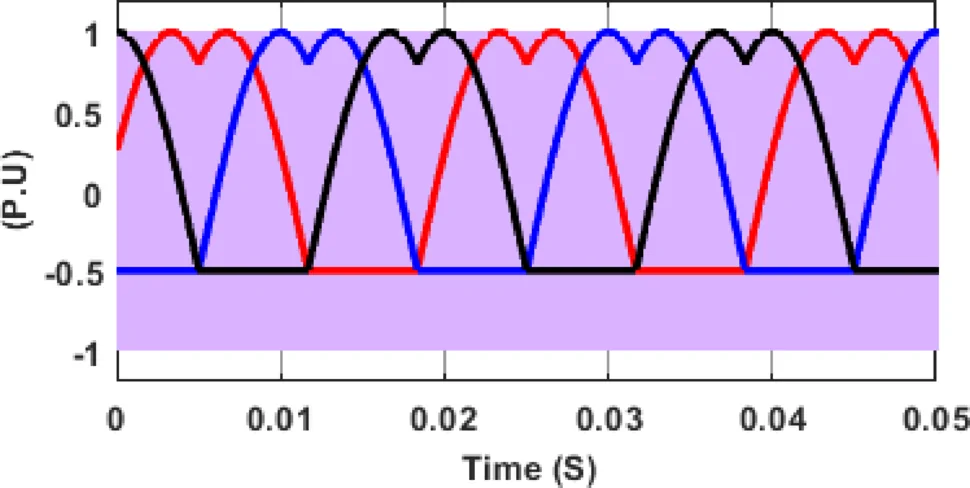
Three-phase modulating signal waveforms for the MSVPWM schemes.

**Fig. 20:**
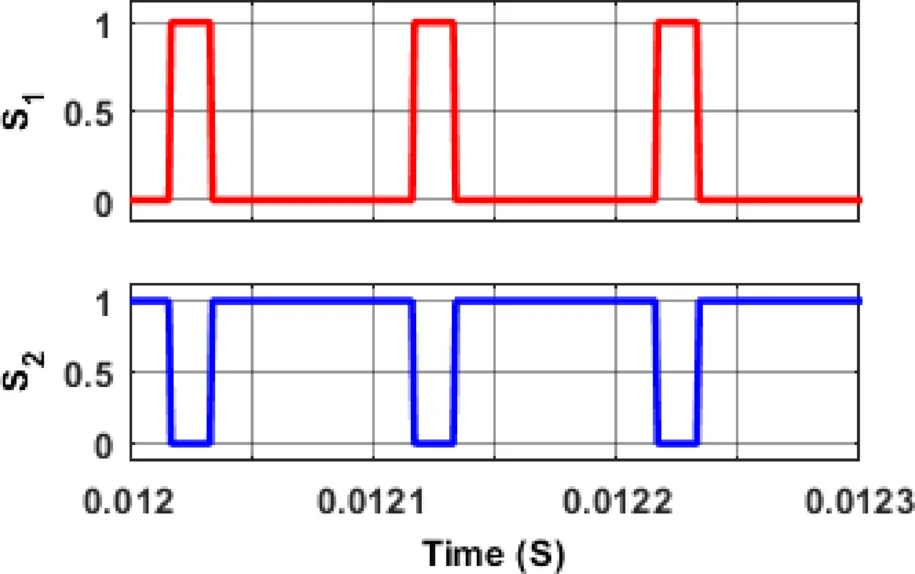
Gate pulse waveform for an SSI leg operating under SVPWM.

**Fig. 21:**
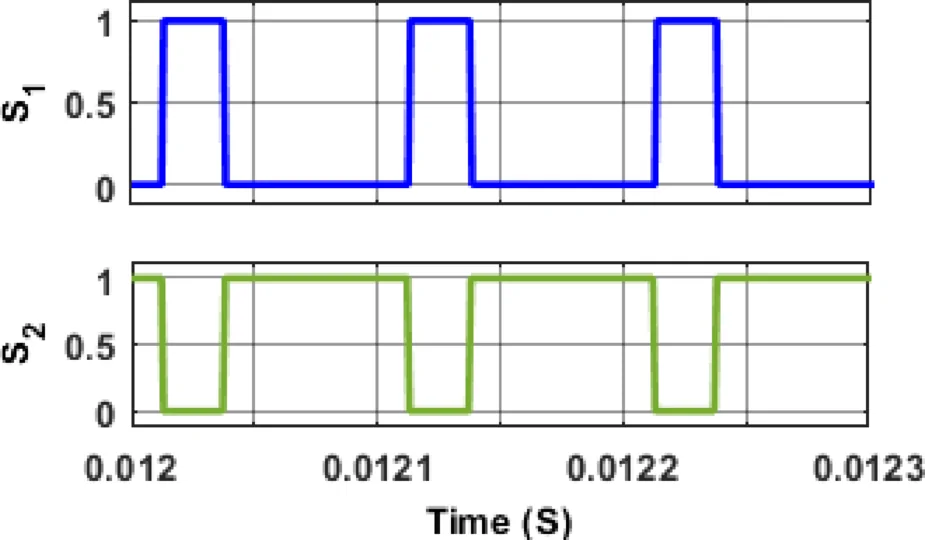
Gate pulse waveform for an SSI leg operating under MSVPWM.

**Fig. 22:**
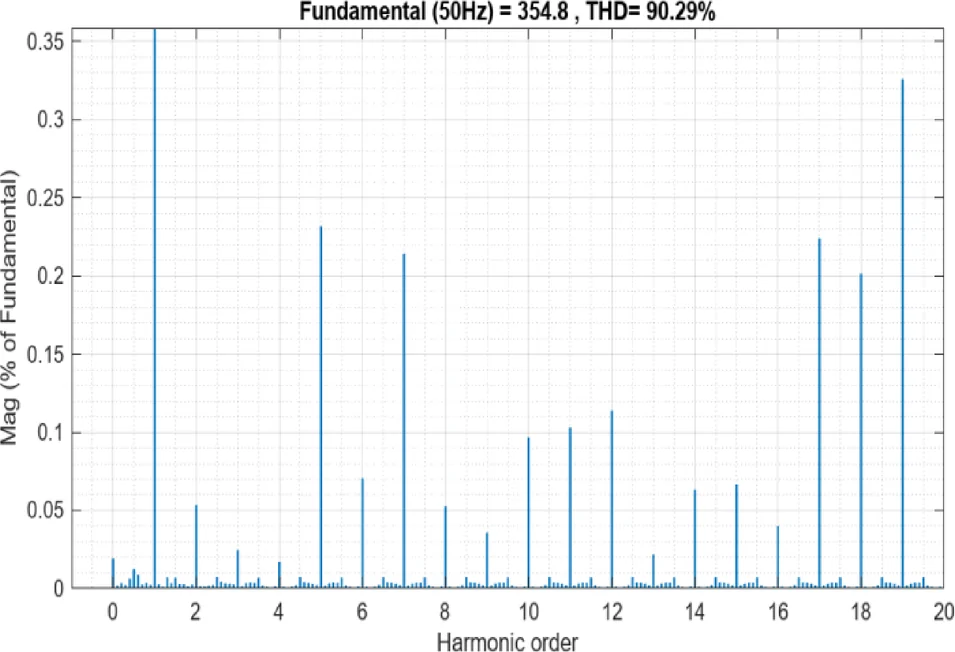
Voltage THD measurement for SVPWM.

**Fig. 23:**
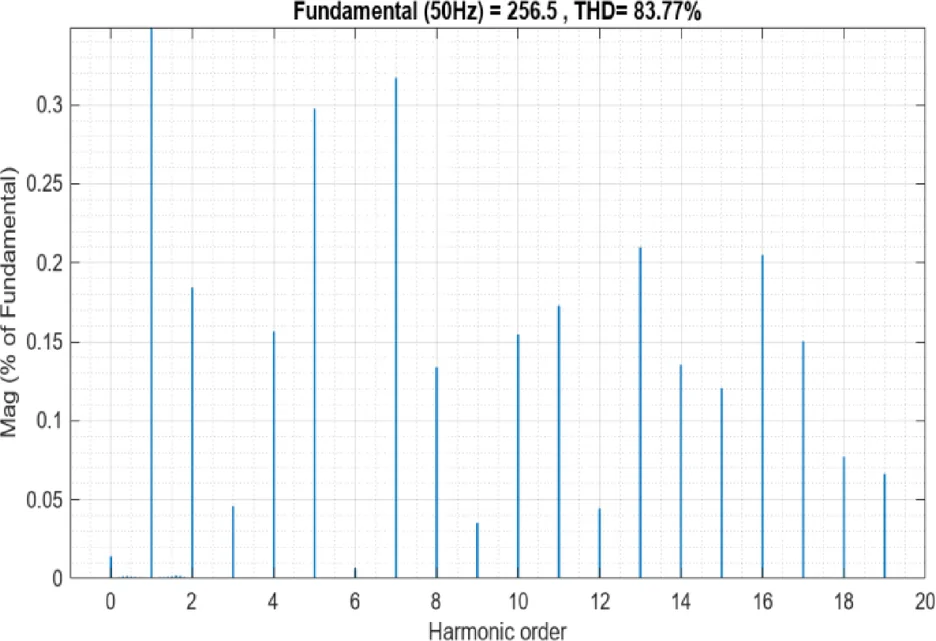
Voltage THD measurement for MSVPWM.

Figures 16 and 17 present the voltage across the diodes, while Figs. 18 and 19 illustrate the reference waveforms for both methods. Figures 20 and 21 depict the gate pulses for leg one of the SSI. Figures 22 and 23 present the THD analysis of the line voltage, offering a quantitative evaluation of waveform quality. The results emphasize the harmonic content of the output and demonstrate the effectiveness of MSVPWM in reducing distortion compared to the SVPWM method.

## Experimental validation

**Fig. 24:**
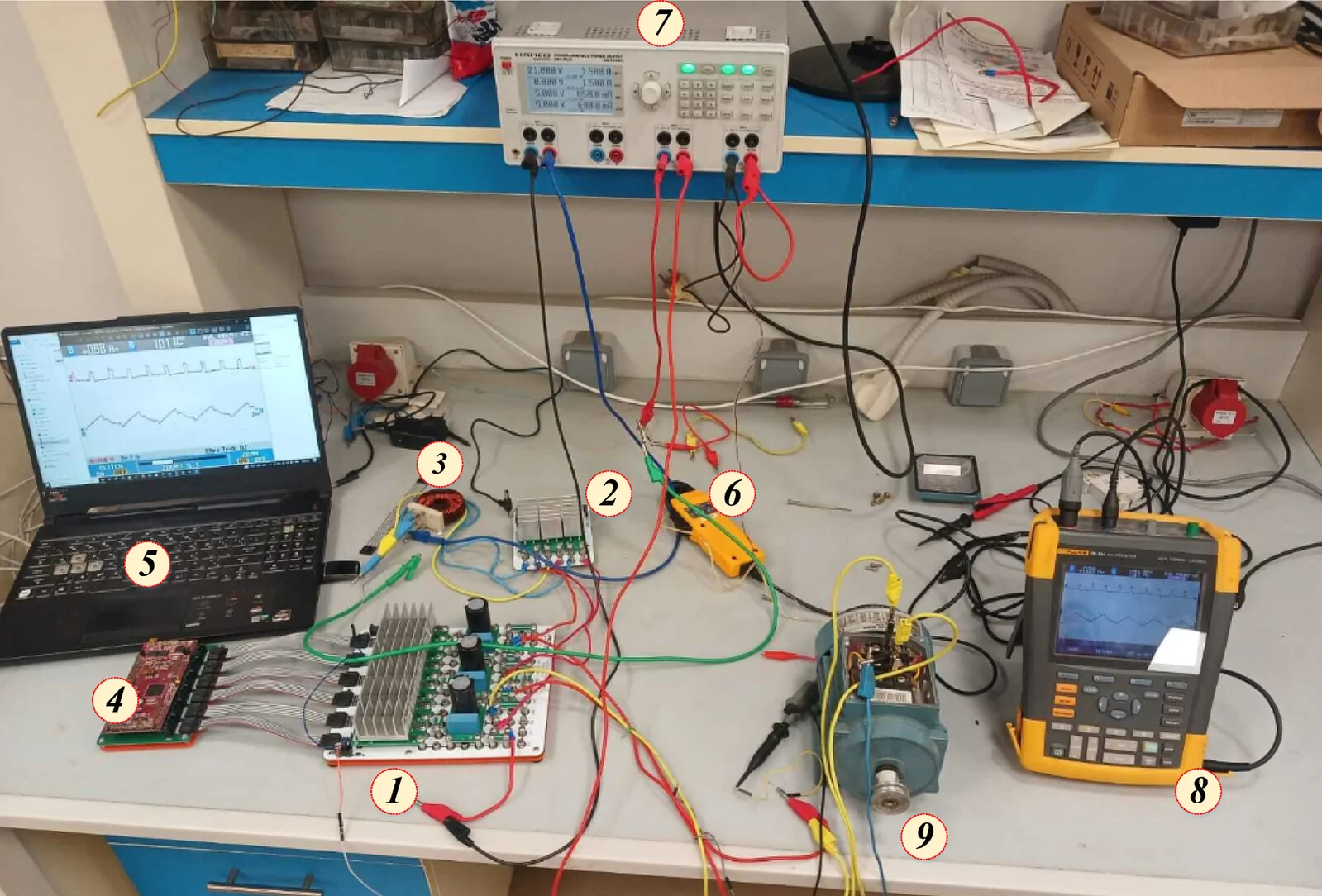
Photographs of the experimental setup. (1)Six IGBT modules, (2) Three diodes Modules (3),The inductor ,(4) F28379D Launchpad Kit Card™ (5), lab top ,(6) current probe (7) Dc supply ,(8) The oscilloscope (9)Induction machine loads.

To validate the performance of the analyzed topology, a prototype of the SSI was designed and implemented. The parameters used in the prototype are detailed in Table 3, with the experimental setup shown in Fig. 24. The SSI prototype was constructed using the Texas Instruments LaunchPad LAUNCHXL-F28379D DSP board, which was utilized to generate gating pulses for Modified Space Vector Modulation (MSVPWM) and featured a dead-time setting of 1.5 µs. The inductor and capacitor values selected for this implementation were 2.5 mH and 150 µF, respectively. This experimental configuration offers a robust platform for comprehensive testing and analysis of the SSI performance. It provides an opportunity to evaluate key operational characteristics, including efficiency, output waveform quality, and dynamic response. Additionally, the insights gained from this experimental setup underscore the potential of the SSI for use in advanced power conversion systems, demonstrating its practicality for real-world applications.

**Table 3:** Experimental study parameters for the SSI.

Parameter	Value	Parameter	Value
Switching Frequency	15 kHz	DSP	TI-F28379D
Dead time	1.5 µs	M	0.8
Inductor	2.5 mH	Load	IM (1 kW)
Capacitor	150 µF/400 V

**Fig. 25:**
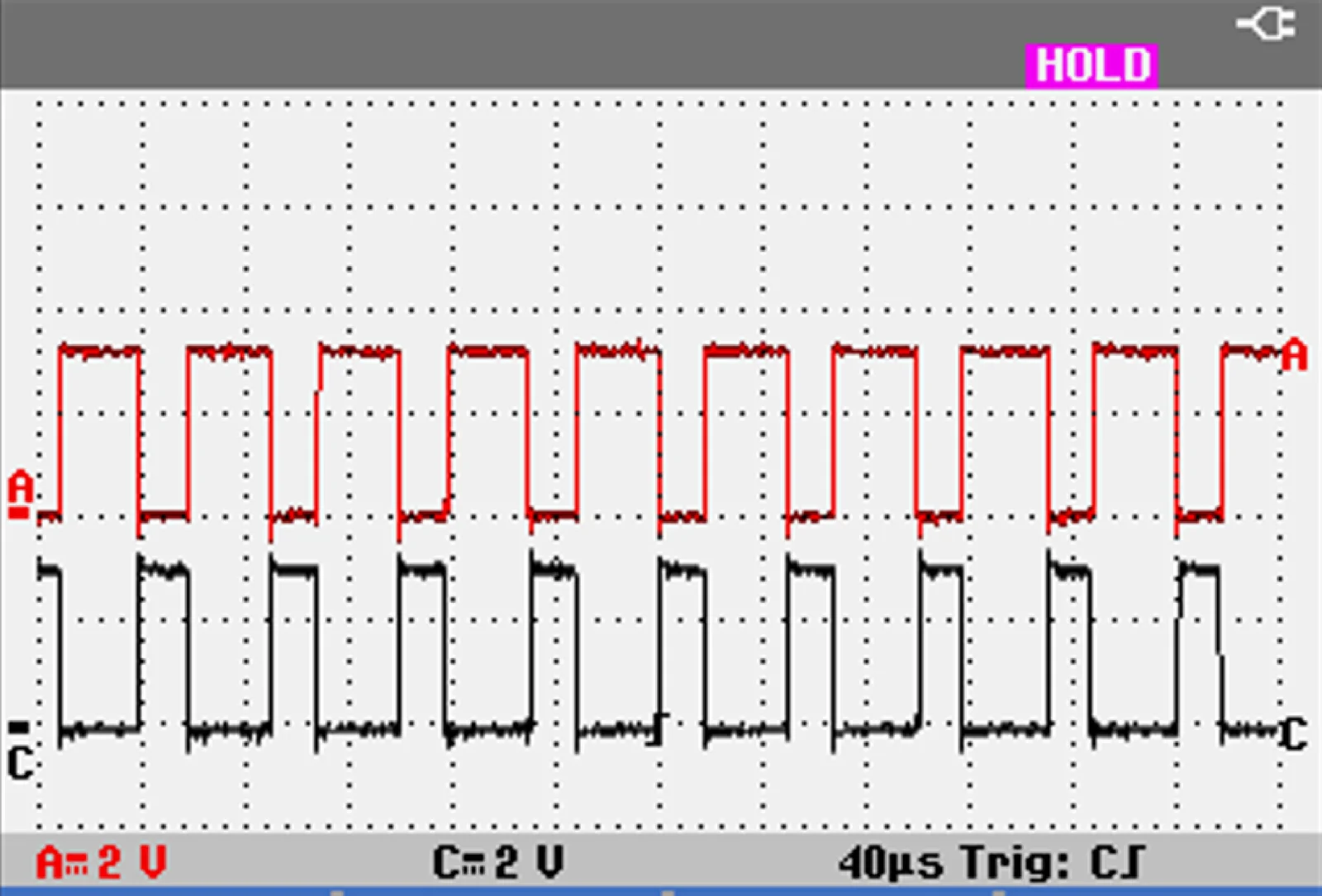
Photograph of the gating pulses of two switches of three-phase legs waveforms.

**Fig. 26:**
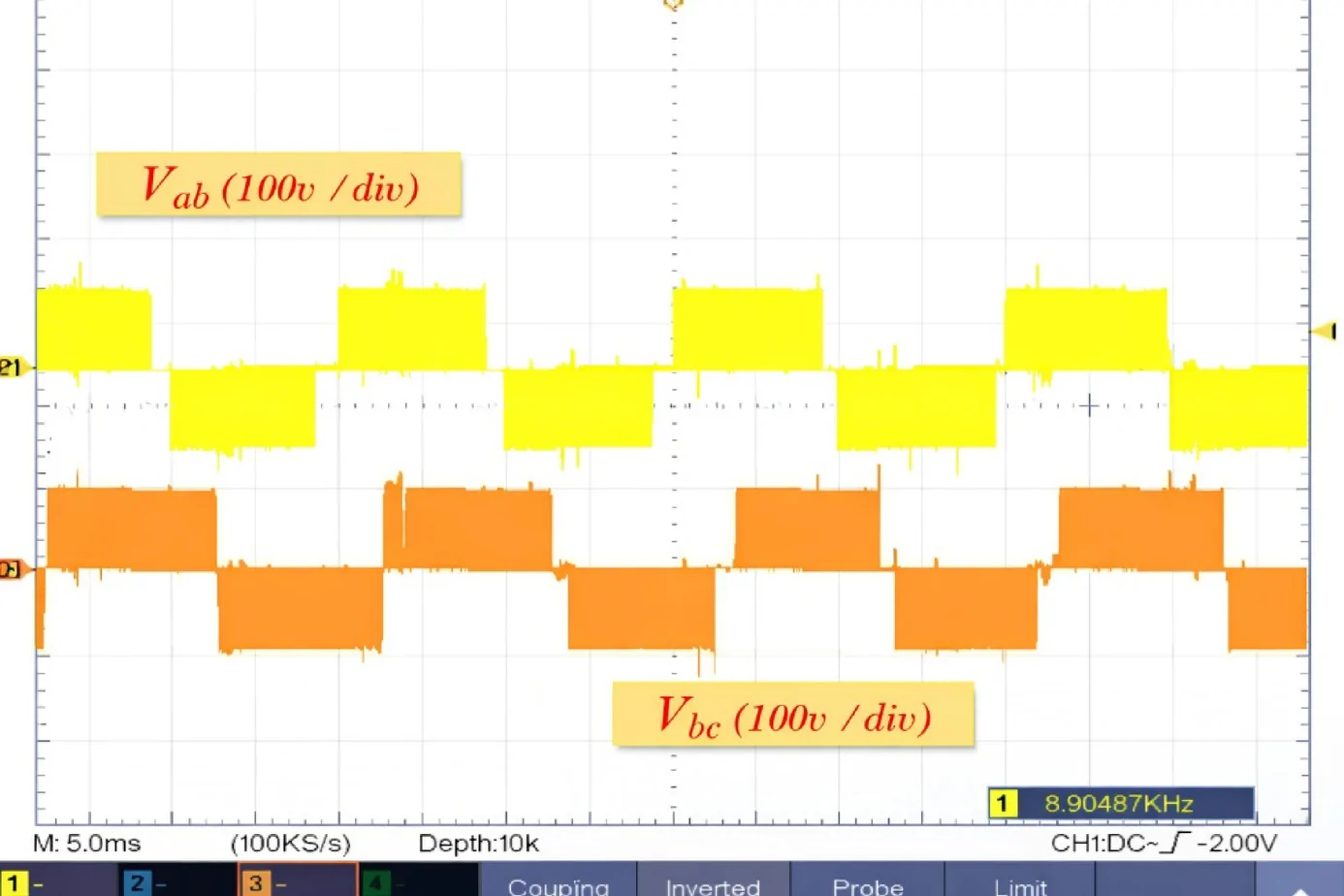
Photograph of the line voltage (${V}_{ab },{V}_{bc}$) waveforms.

**Fig. 27:**
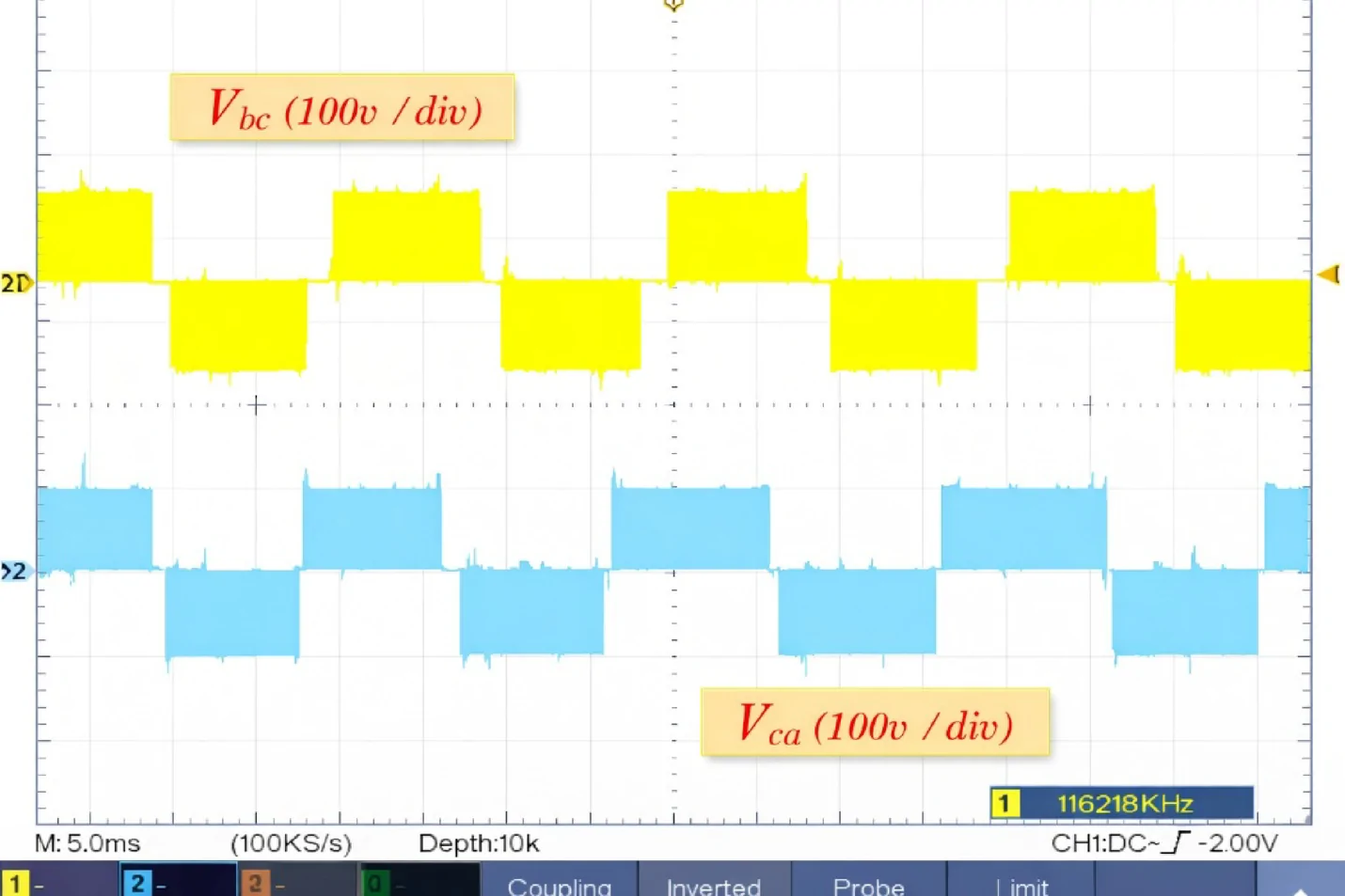
Photograph of the line voltage (${V}_{bc }, {V}_{ca}$) waveforms.

Figure 25 shows the gating pulses of two switches belonging to different legs of the three-phase inverter. The pulses are well defined, with proper dead-time insertion to avoid shoot-through, thereby ensuring safe operation of the switching devices. The consistent and symmetrical switching patterns highlight the correct implementation of the MSVPWM strategies on the F28379D Launchpad controller. These signals provide the foundation for generating high-quality output voltages and balanced three-phase currents. Figures 26 and 27 illustrate the measured line voltages ${V}_{ab },{V}_{bc } ,an {V}_{ca}$, respectively. The results demonstrate nearly sinusoidal voltage waveforms with the expected peak amplitude and phase displacement. The smoothness and periodicity of the signals confirm that the inverter successfully synthesizes balanced three-phase voltages with minimal harmonic distortion. This validates the accuracy of the modulation strategy in achieving sinusoidal outputs suitable for sensitive motor and grid applications.

**Fig. 28:**
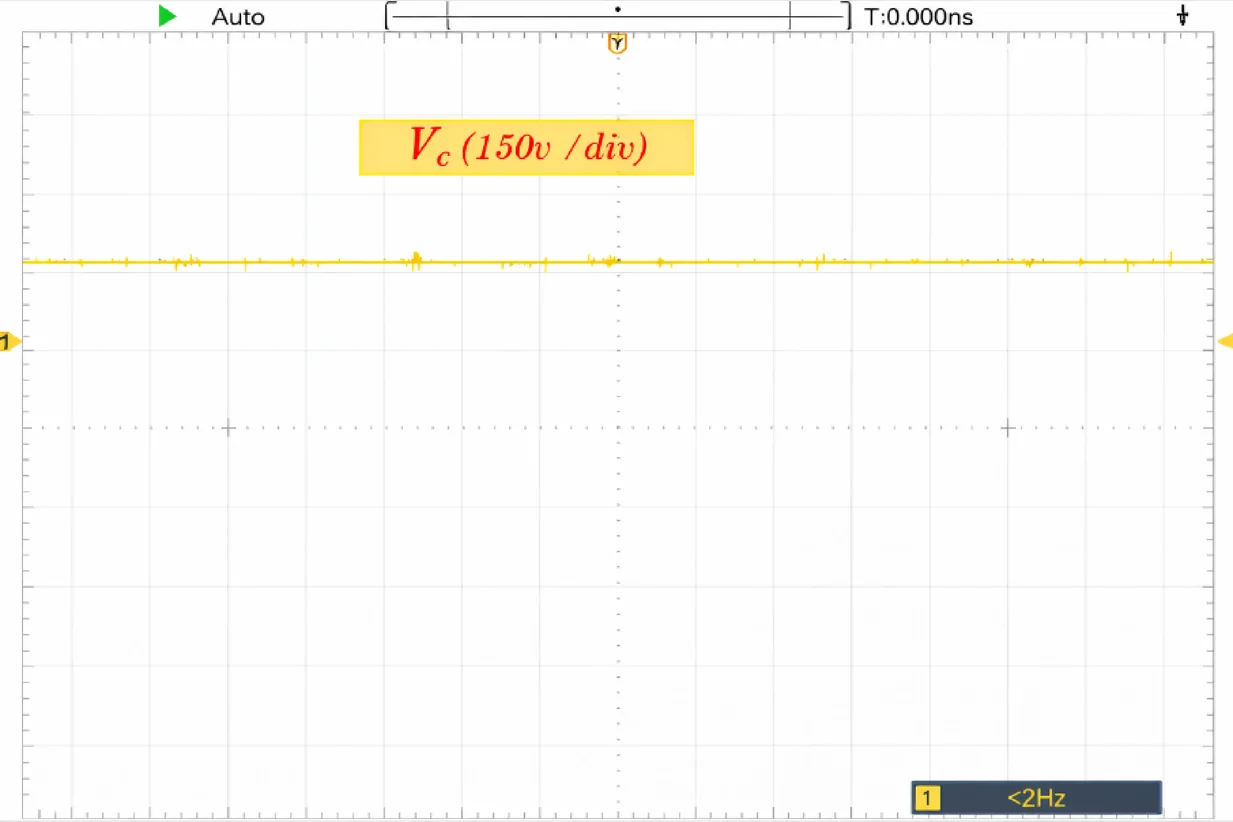
Photograph of the voltage across *C* waveform.

Figure 28 presents the voltage across the DC-link capacitor *C*. The waveform remains stable with minimal ripple, demonstrating the capacitor effectiveness in filtering voltage fluctuations and maintaining a steady DC bus voltage for the inverter. The small observed ripples are within acceptable limits, confirming proper capacitor sizing and its role in ensuring uninterrupted energy supply during dynamic load conditions.

**Fig. 29:**
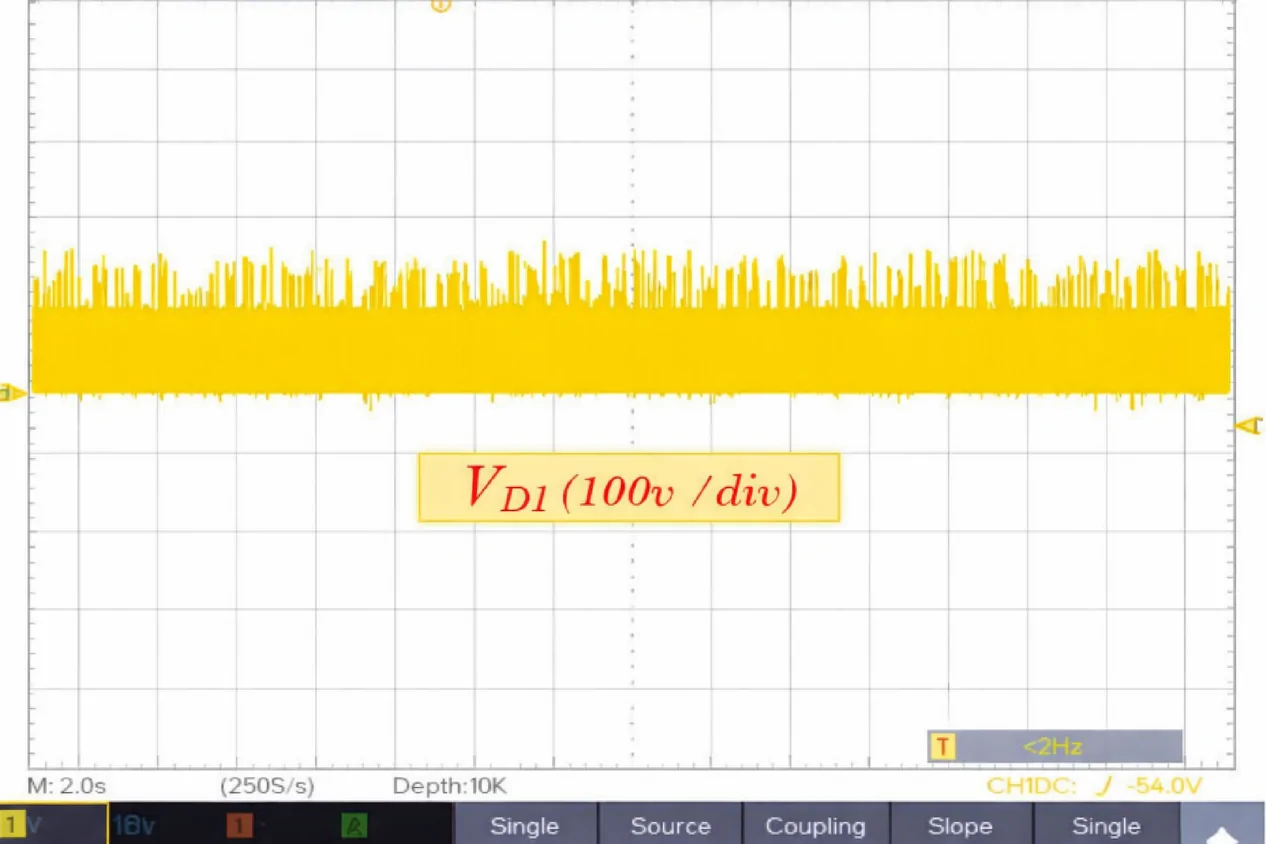
Photograph of the voltage across *L* waveform.

Figure 29 depicts the voltage across the input inductor *L*. The waveform shows smooth transitions and low ripple content, indicating that the inductor is efficiently regulating the current flow into the inverter stage. This highlights the inductor function in suppressing high-frequency switching noise, limiting current stress on semiconductor devices, and ensuring stable energy transfer between the source and load.

**Fig. 30:**
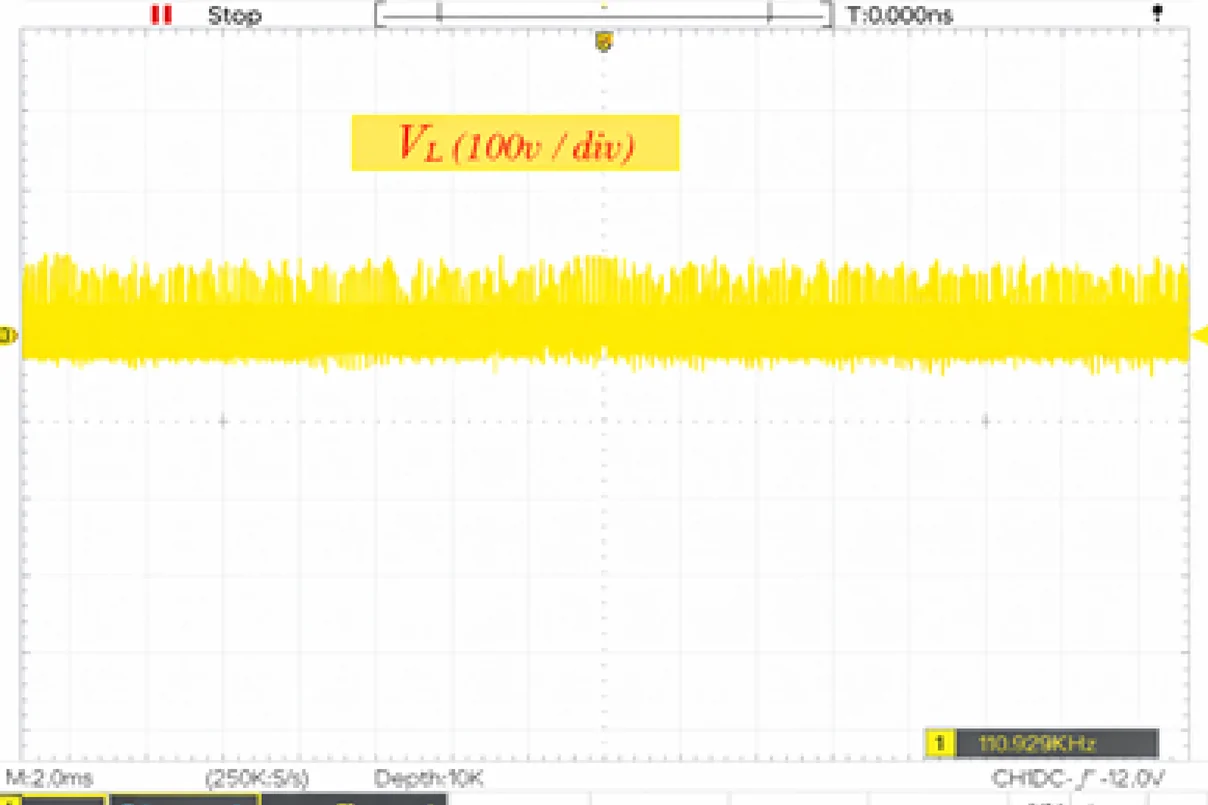
Photograph of the voltage across *D*_1_ waveform.

**Fig. 31:**
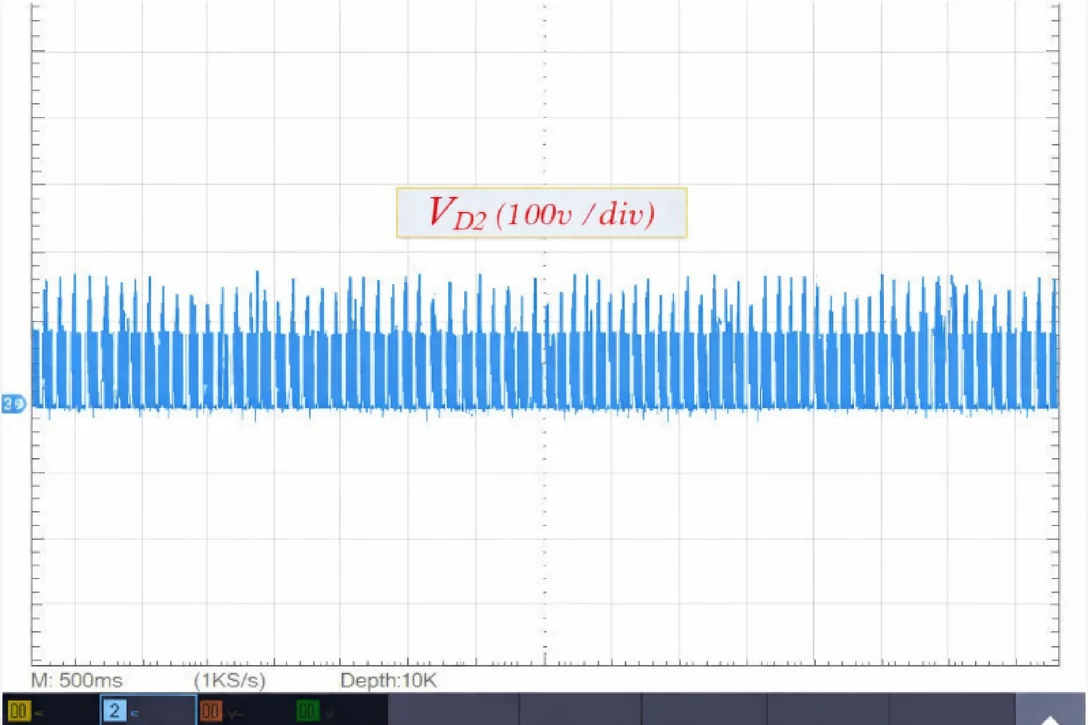
Photograph of the voltage across *D*_2_ waveform.

**Fig. 32:**
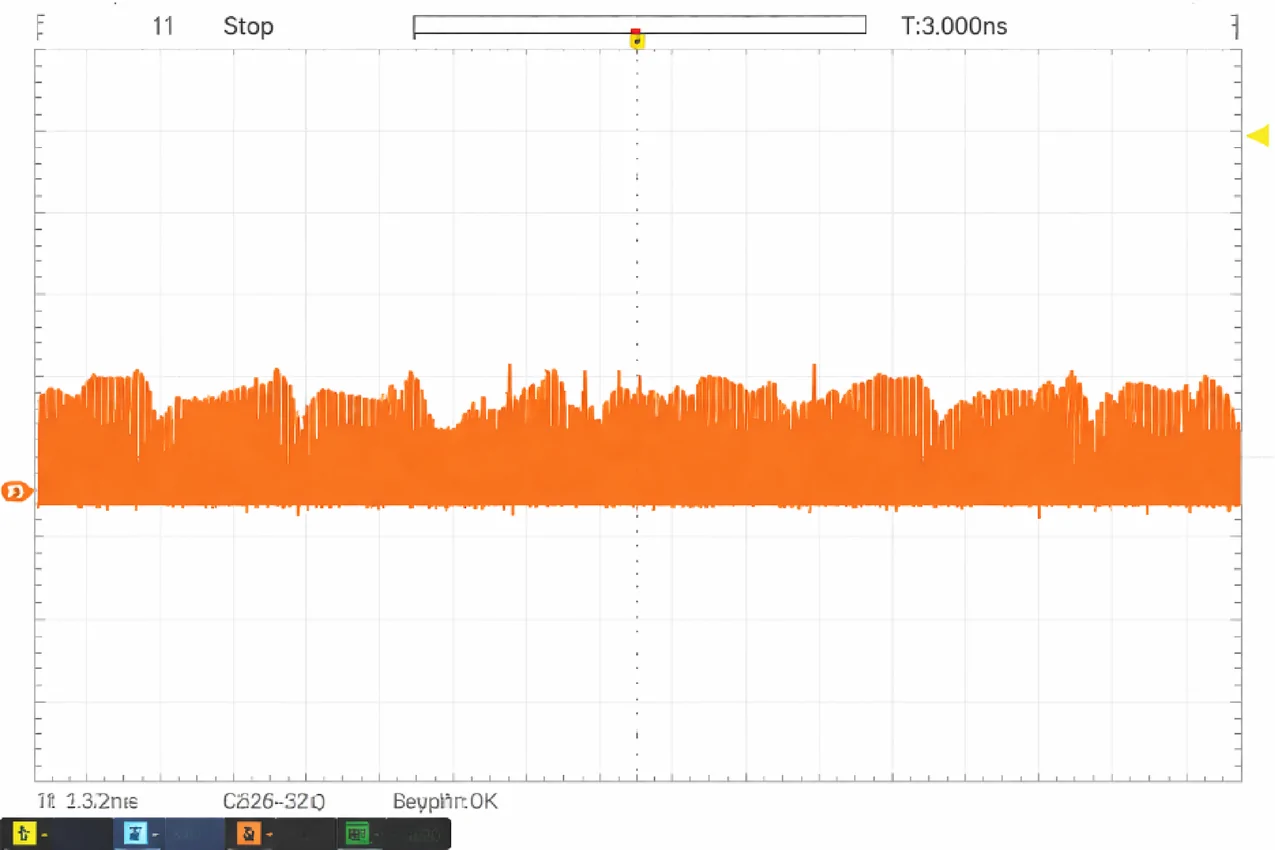
Photograph of the voltage across *D*_3_ waveform.

Figures 30, 31, and 32 display the voltage stresses across diodes ${D}_{1 },{D}_{2} , and {D}_{3}$​ respectively. The captured waveforms confirm proper diode commutation during switching events, with voltage excursions staying within rated limits. This not only validates the safe design margin of the selected diodes but also demonstrates the reliability of the proposed topology under real operating conditions.

**Fig. 33:**
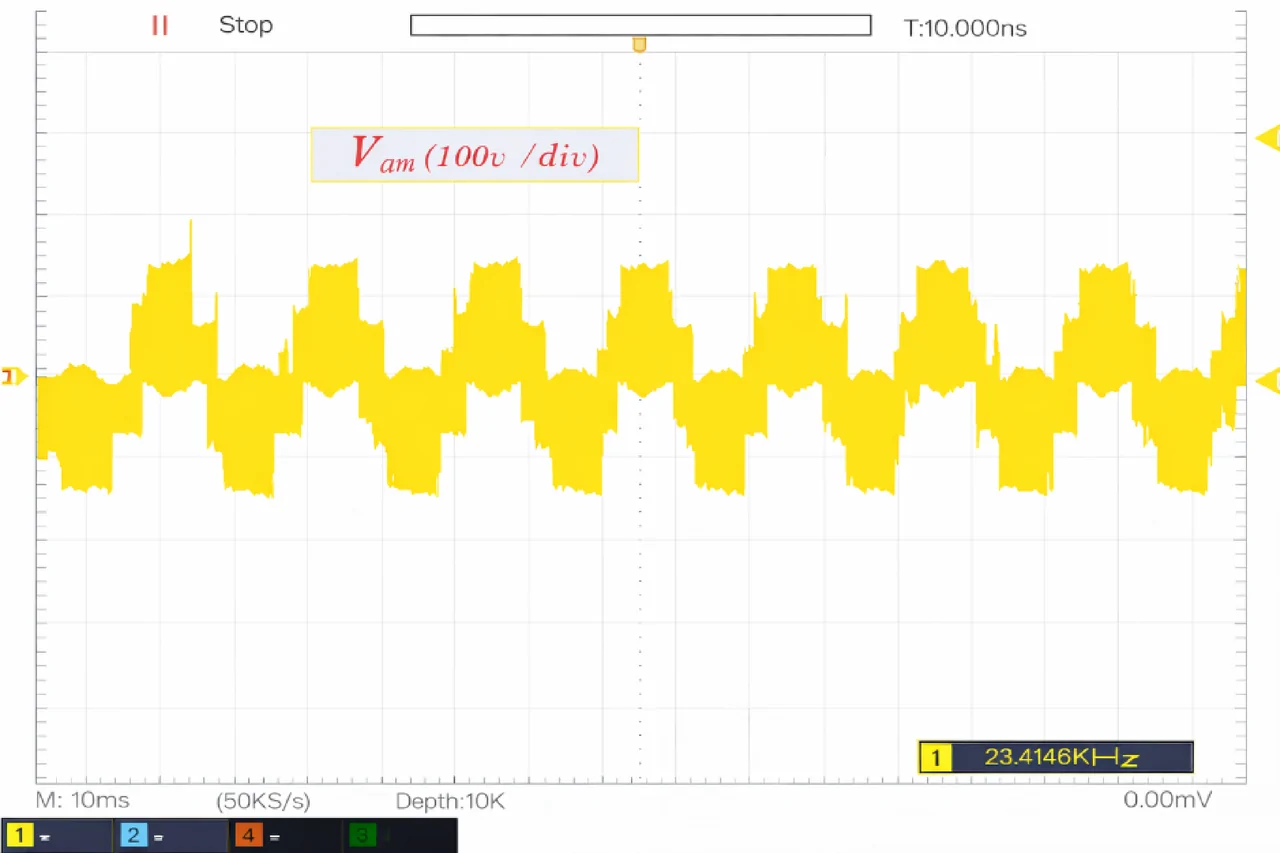
Photograph of the phase voltage (*V*_an_) waveform.

Figure 33 shows the phase voltage waveform ${Van}$​. The captured signal exhibits a sinusoidal shape that closely matches the theoretical reference waveforms. The absence of significant distortion confirms the inverter capability to produce high-quality phase voltages, which is critical for driving three-phase induction machines with minimal torque ripple and improved efficiency.

**Fig. 34:**
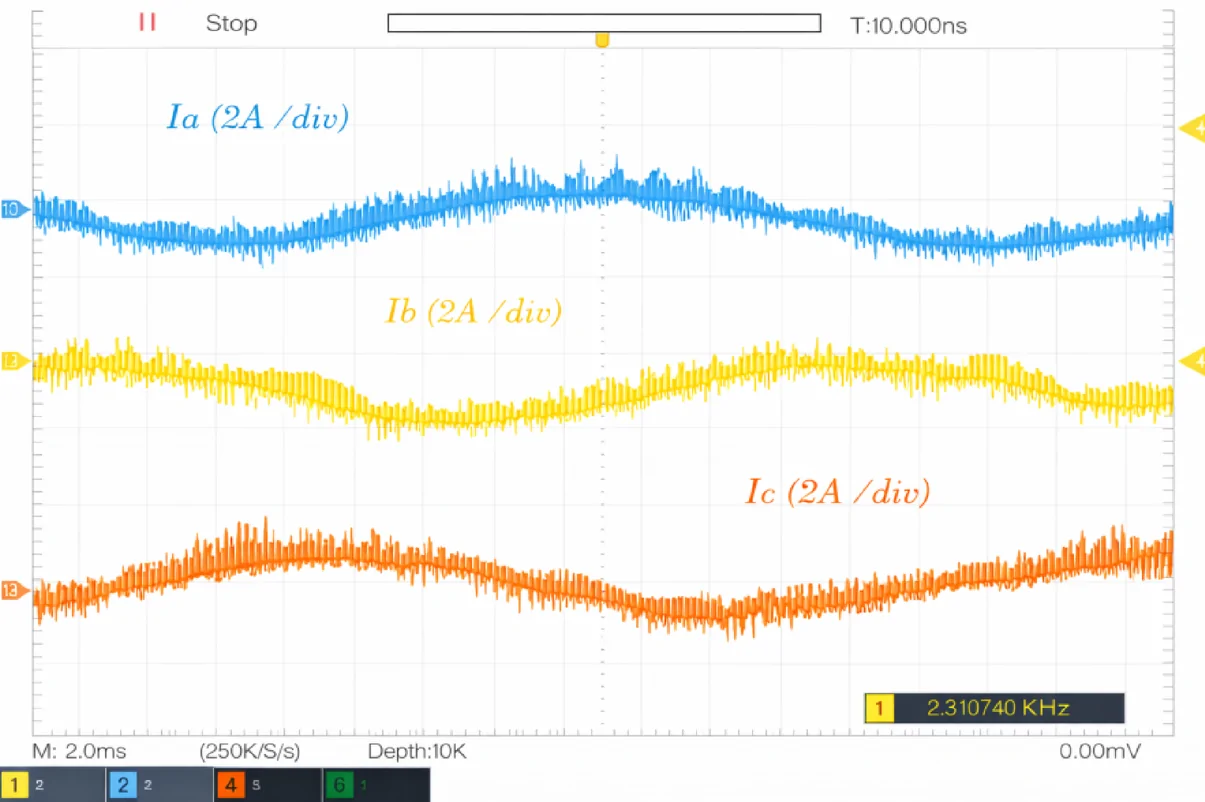
Photograph of the three-phase load current (*i*_*abc*_) waveforms.

Figure 34 presents the three-phase load current waveforms (${I}_{a },{I}_{a }, \mathrm{and} {I}_{c} )$.The currents are well balanced, sinusoidal, and exhibit a 120° phase separation, validating that the inverter delivers symmetrical three-phase outputs. The smooth and uniform behavior of the load currents confirms the system ability to drive induction machine loads effectively while maintaining a high-power factor and low harmonic distortion.

**Fig. 35:**
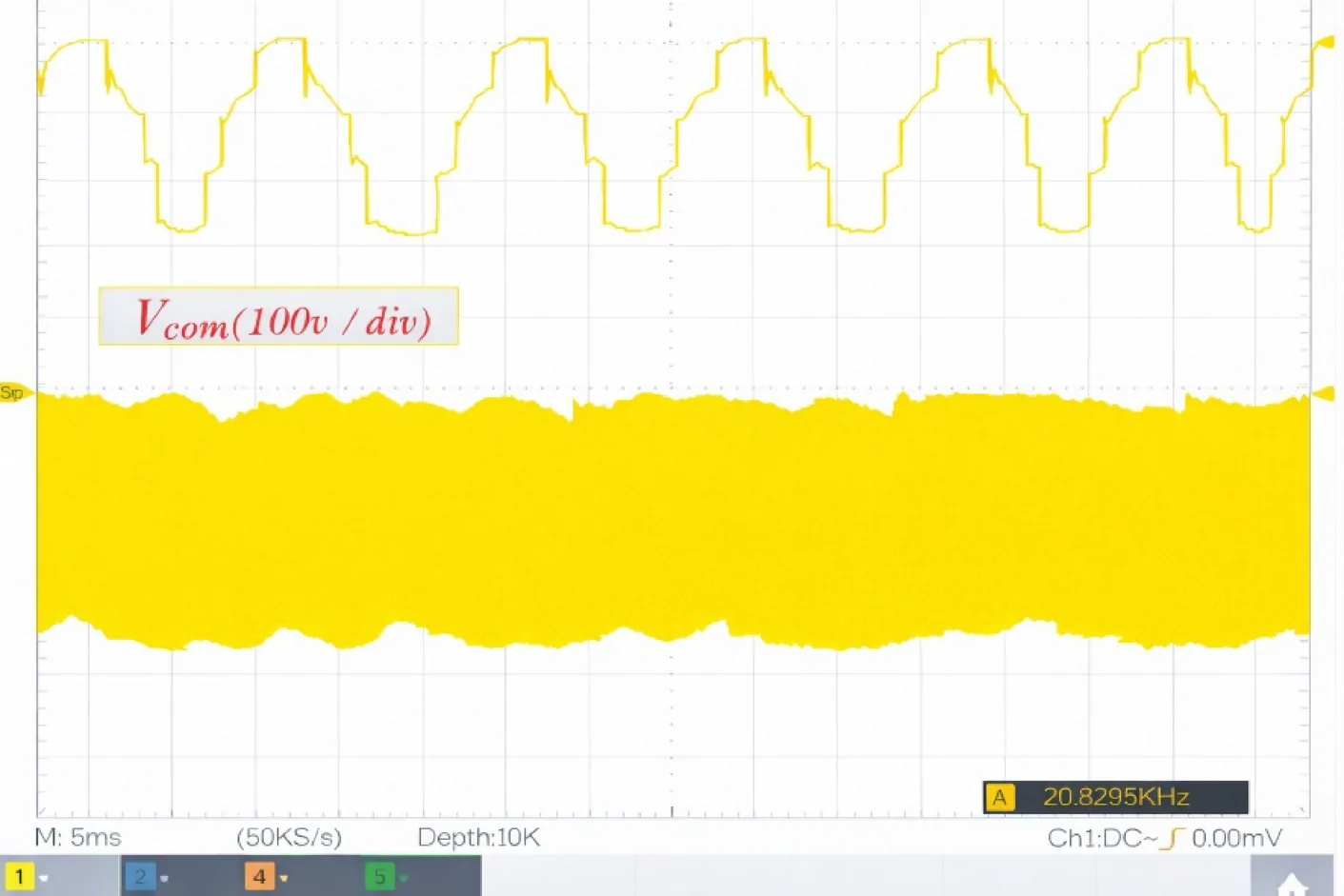
Photograph of the CMV waveform of the SSI under SVPM.

**Fig. 36:**
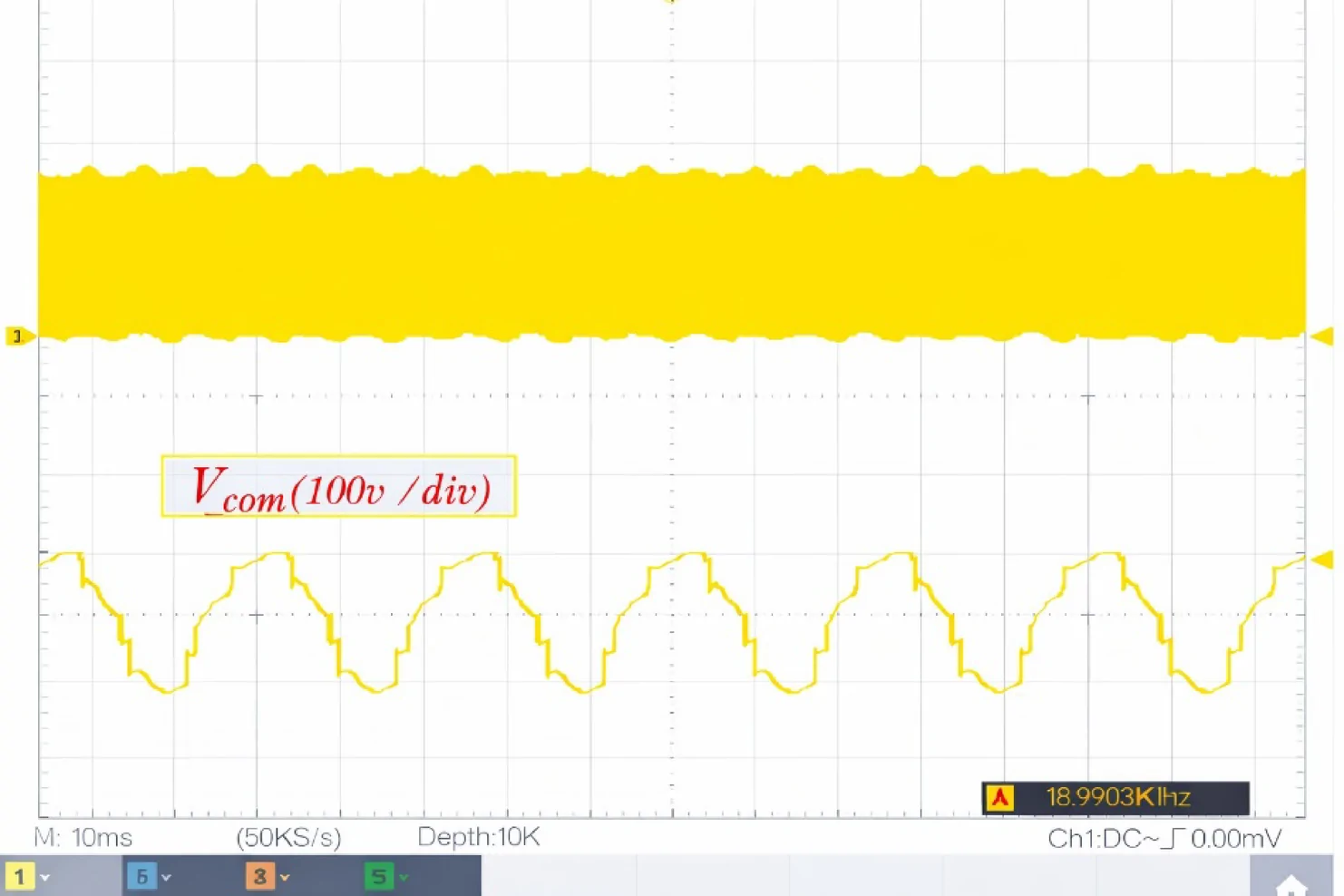
Photograph of the CMV waveform of the SSI under MSVPM.

**Fig. 37:**
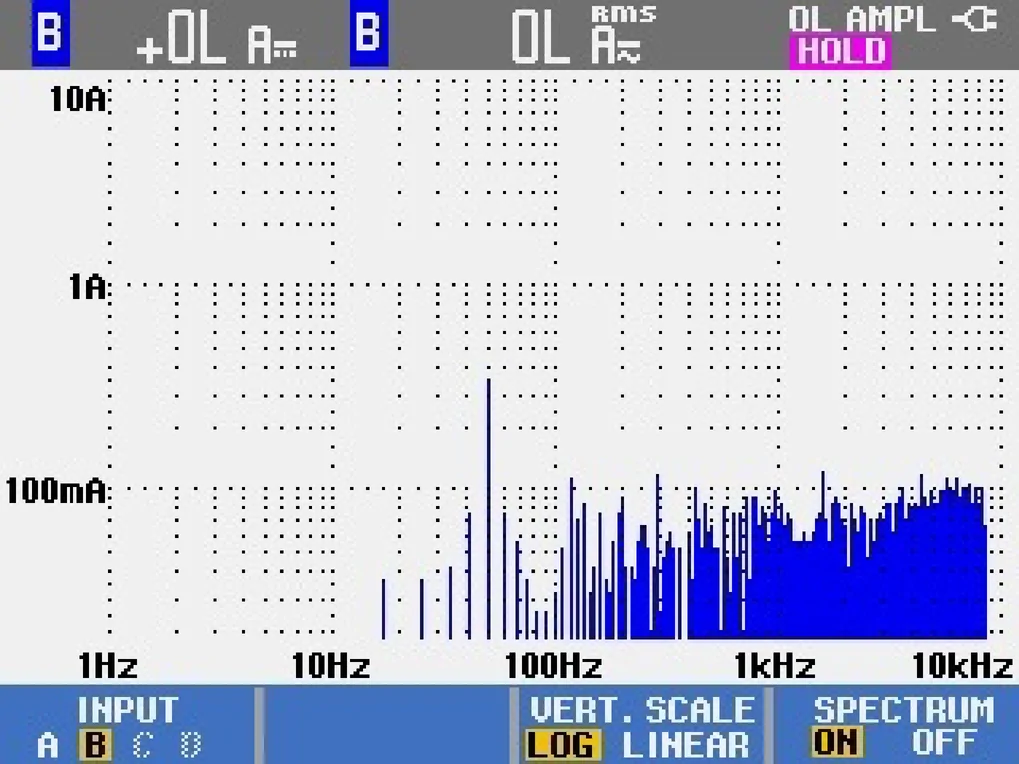
Voltage THD.

**Fig. 38:**
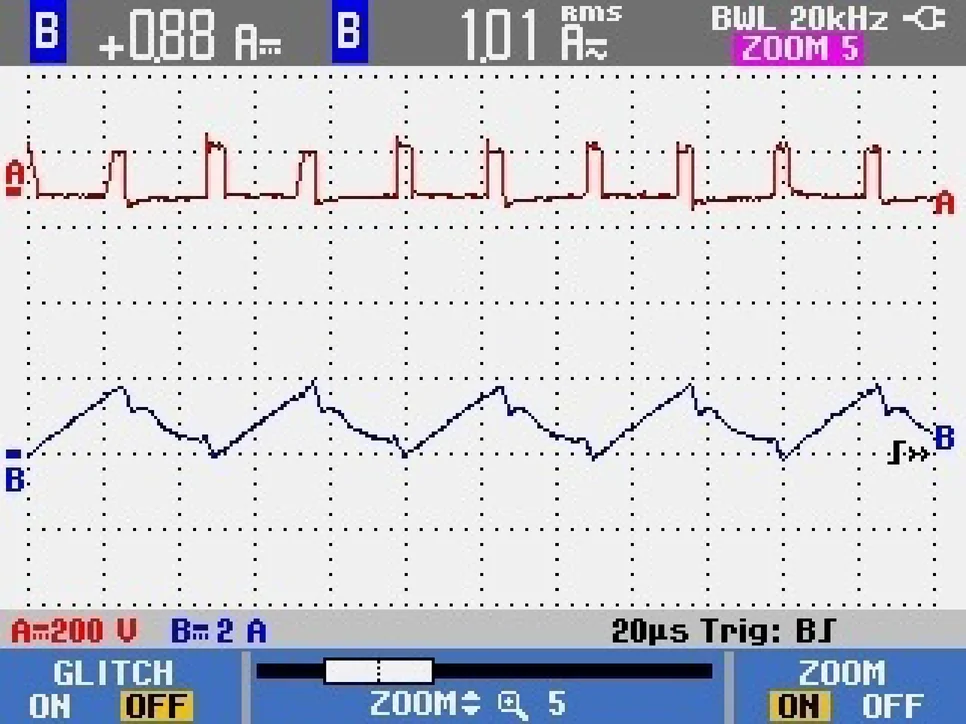
Inductor current waveform.

**Fig. 39:**
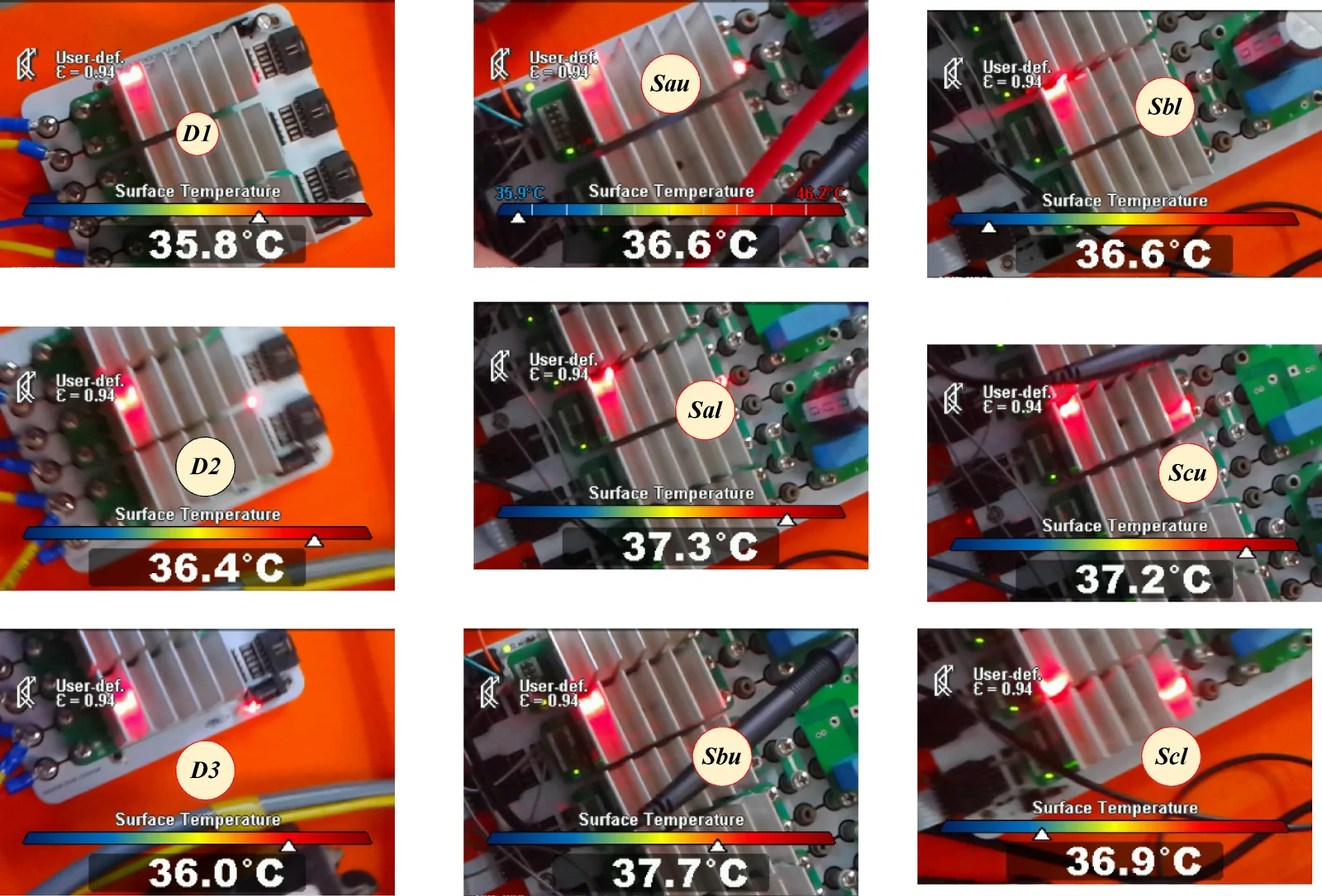
The temperature measurements of three diodes.

Figures 35 and 36 compare the CMV waveforms of the SSI under SVPWM and MSVPWM. The results show that while SVPWM produces relatively higher CMV amplitudes, MSVPWM achieves a significant reduction in CMV magnitude. This is particularly important in reducing leakage currents and electromagnetic interference (EMI), thereby improving overall inverter reliability and extending the lifespan of motor windings and insulation. The reduction in CMV further demonstrates the superiority of MSVPWM over conventional SVPWM in practical applications. The THD spectrum shown in Fig. 37 the frequency components of the inverter output signal. The x-axis displays the frequency on a logarithmic scale from 1 Hz to 10 kHz, while the y-axis represents the amplitude of each component, also on a logarithmic scale. The fundamental frequency component is the dominant peak at the lower frequency range, corresponding to the main operating frequency of the inverter. Smaller peaks at integer multiples of the fundamental indicate harmonic components, which arise from the non-linear switching behavior of the inverter. Most harmonic amplitudes are relatively low compared to the fundamental, suggesting that the total harmonic distortion (THD) is small and the output waveform is of high quality. The higher-frequency components, observed between 1 and 10 kHz, are attributed to PWM switching and are typical in inverter outputs. Overall, the spectrum demonstrates that the proposed inverter maintains a low THD, confirming effective waveform control and minimal distortion. Figure 38 illustrates the charging and discharging of the inductor current. Figure 39 illustrates the temperature profiles of the three diodes and six IGBT switches during operation. The results demonstrate that the implemented thermal management system successfully maintains all device temperatures within safe operating limits, thereby enhancing system reliability and prolonging the overall lifetime of the power module.

The experimental results collectively validate the theoretical analysis and simulation findings. The gating signals confirm the correct application of modulation techniques, while the measured voltages and currents demonstrate the inverter ability to deliver high-quality, sinusoidal, and well-balanced outputs. The capacitor and inductor voltage profiles emphasize the robustness of the passive components in stabilizing energy flow and suppressing ripple. Moreover, the diode waveforms confirm safe commutation and appropriate device selection.

## Conclusions

This study introduced a robust and efficient modulation strategy for improving the performance of Split-Source Inverters (SSIs) in PV-based applications, with a particular focus on solar-powered water pumping systems. The proposed Space Vector Modulation (SVM) and Modified Space Vector PWM (MSVPWM) methods effectively mitigate high-frequency common-mode voltage (CMV), reducing leakage currents and EMI while preserving the essential voltage boosting and inversion functions of the SSI topology. Simulation and experimental results confirm the effectiveness of the approach, achieving a 7.6% reduction in THD, a 66.7% reduction in CMV, and accurate tracking of three-phase voltages and currents to ensure high power factor performance. The single-stage SSI architecture unifying boosting and inversion provides significant advantages in component count reduction, energy conversion efficiency, and control simplicity. Furthermore, the inverter has been theoretically validated for medium-voltage and high-power applications, and all simulation results have been verified experimentally under various loads and transient conditions, including modulation index, DC-link voltage, frequency variations, and resistive/inductive load transitions, the proposed inverter demonstrates robust, efficient, and scalable operation, making it a highly practical and competitive alternative to conventional two-stage inverters in PV and renewable energy systems.

## Data Availability

The original contributions presented in the study are included in the article, further inquiries can be directed to the corresponding author.
